# Polyimide Polymer Simulations through Coarse-Grained Modeling: Prediction of Structure, Physical Properties, and Gas Separation Properties

**DOI:** 10.1021/acs.jpcb.4c04595

**Published:** 2025-04-30

**Authors:** Amro M. O. Mohamed, Ioannis G. Economou, Hae-Kwon Jeong

**Affiliations:** † Chemical Engineering Program, Texas A&M University at Qatar, P.O. Box 23874, Doha 122104, Qatar; ‡ Artie McFerrin Department of Chemical Engineering, Texas A&M University, 3122 TAMU, College Station, Texas 77843-3122, United States; § Department of Materials Science and Engineering, Texas A&M University, 3122 TAMU, College Station, Texas 77843-3122, United States

## Abstract

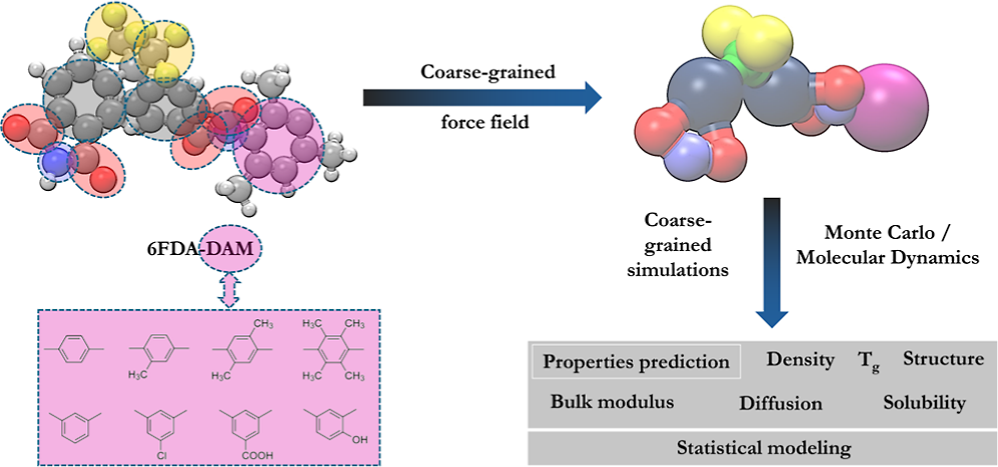

In this study, we introduce a set of coarse-grained (CG) force field parameters for simulating a series of 6FDA-based polyimides. Utilizing atomistic descriptors, we developed CG models that accurately predict the specific volume of the polymers under investigation. Our findings suggest that certain parameters, particularly those associated with specific diamines, can be employed to predict properties such as density using a multiple linear regression. Our study further explores the halogenation of diamines and proposes methods for estimating intermolecular interaction parameters. Our calculations refer to various structural properties, including the radius of gyration, end-to-end distance, glass transition temperature, and diffusion coefficients. Utilizing the newly developed CG force field parameters, we conducted gas separation simulations for 6FDA-DAM polyimide, particularly to predict both sorption- and diffusion-separation mechanisms within the polymer. These simulations provided excellent agreement with experimental data on solubility, diffusion, and permeability selectivity for CO_2_/CH_4_, O_2_/N_2_, and propylene/propane. The results contribute significantly to our understanding of polyimide behavior, and the parameters proposed here offer a promising tool for the development of new materials with tailored properties for targeted applications.

## 1 Introduction

In recent years, polyimide (PI) polymers have gained significant attention for their use in gas separation applications due to their exceptional thermal and chemical stability, mechanical strength, and tunable permeability.^1−3^ PIs have demonstrated high-performance gas separation capabilities, particularly for CO_2_/N_2_ and CO_2_/CH_4_ separation, which are essential for carbon capture and storage, natural gas purification, and biogas upgrading.^4,5^ The high free volume and selective transport channels within the PI matrix are crucial for achieving high gas permeability and selectivity in membranes, while the functional groups on the PI backbone contribute to the solubility properties and facilitate gas diffusion.^6^

Mixed-matrix membranes (MMMs), combining the advantages of polymeric and inorganic materials, have emerged as promising materials to enhance gas separation performance.^7^ PIs have been utilized as the continuous matrix in MMMs, where their compatibility with inorganic fillers [such as metal–organic frameworks (MOFs), zeolites, and carbon nanotubes] has been shown to improve the overall gas separation efficiency.^6,8−10^ Additionally, PI-based hybrid membranes have demonstrated an enhanced gas separation performance through the incorporation of protic ionic liquids (ILs). The ILs, confined within the PI matrix, decreased the fractional free volume and narrowed gas transport channels, resulting in a remarkable increase in NH_3_/CO_2_ selectivity.^11^ These advancements highlight the potential of PI-based MMMs and hybrid membranes for addressing gas separation challenges in various industries.

Molecular simulation and modeling have become powerful tools in material science and engineering for predicting the structure of polymers and other materials, and gas separation properties. Researchers have used molecular simulation to study the effect of various factors on the gas separation properties of polymers, including temperature, pressure, and polymer chain length.^12−14^ Molecular dynamics (MD) simulations have allowed researchers to study the behavior of polymer chains at a molecular level.^15−17^ Atomistic models have been highly successful in providing detailed insights on the structure and prediction of properties of glassy polymers.^18^ The accurate representation of molecular interactions at the atomistic level has enabled the precise calculation of key properties such as various thermodynamic properties including solubility, penetrant-induced plasticization,^19,20^ and glass transition temperature.^21^ However, atomistic modeling of glassy polymers faces several significant challenges.^18,22^ One major challenge is the computational demand associated with simulating high-molecular-weight polymers or performing extended simulations over long timescales. Glassy polymers often exhibit slow dynamics with very long characteristic times, making it difficult to capture long-term behaviors and transitions using atomistic simulations. In addition, limitations in computational resources make it challenging to assess large polymer systems or investigate complex phenomena such as gas diffusion and mechanical deformation. Also, atomistic models rely on empirical force fields, which require fine-tuning to align precisely with experimental data.^18^ Glassy polymers are promising for various gas separation applications, particularly when incorporated into organic–inorganic membranes. The mechanical properties and interaction parameters of these membranes are crucial for their performance in such applications, but these aspects are difficult to study comprehensively using solely atomistic simulations.^23^

Previous research has shown that a well-validated set of coarse-grained (CG) models can provide valuable insights into the structure and properties of various types of polymers, including polyethylene and polypropylene,^24^ polystyrene,^25^ and PI.^26^ CG MD simulations have been widely used to study various aspects of PI systems, such as mechanical responses,^27−29^ dynamic properties,^26^ and piezoelectric polymer systems.^30^ Sudarkodi et al. demonstrated the capability of CG models to predict differences in mechanical responses between two competing molecular architectures.^27^ Wen et al. developed a CG model of branched poly(ether imide) that reproduced accurately thermal expansion properties and mechanical moduli at different temperatures.^29^ Hu et al. used CG simulation to predict the mechanical properties of cross-linked epoxy polymers.^28^ Pandiyan et al. investigated the effect of different levels of coarse-graining on the stress–strain responses and dynamic properties of high-temperature HFPE-30 PI and found that a model with eight beads, to represent a single monomer, provided more accurate results compared to an all-atom representation.^26^ Chakrabarty and Cagin developed a CG model for piezoelectric PI systems that effectively described various properties, including mechanical, dielectric, and thermal properties while achieving a significant reduction in computational time and an increase in the simulated system size.^30^ Volgin et al. examined the structural and dynamic properties of R-BAPB/C60 nanocomposites using CG MD simulation, highlighting the importance of understanding the degree of chemical heterogeneity on subdiffusive nanoparticle dynamics in polymer nanocomposites.^31^ CG MD simulations were implemented, accompanied by atomistic simulations, for modeling transport phenomena of large penetrants in polymeric systems.^32^ This body of research underscores the potential and versatility of CG models for studying a broad range of physical phenomena and applications of polymers.

CG models have been developed to predict the self-assembly behavior of block copolymers.^33^ The use of molecular simulation has also allowed the prediction of gas permeability in polymer membranes, such as those used in gas separation processes.^34^ The development of accurate force fields has been crucial for simulating the behavior of polymers, and researchers have used a combination of experimental and computational techniques to develop these force fields.^35,36^ In recent years, machine learning techniques have also been introduced to polymer science, allowing for the development of predictive models for polymer properties.^37−39^ Overall, molecular simulation and modeling have become invaluable tools for understanding and predicting the behavior of polymers and will continue to play an important role in the field of polymer science and engineering.

Although atomistic molecular simulations on polymers can accurately predict certain properties, CG models reduce computational cost, enabling mesoscale simulations. In this work, we attempt to overcome the limitation of CG models being able to provide limited transferability across different conditions. Especially with Martini 3 tiny beads, preservation of more chemical details is possible, which allows for better transferability between polymers with variable functionality.^40^ The chemical details are important to correctly predict adsorption and diffusion properties in polymeric systems.^41^ The Martini CG model offers several advantages over other CG techniques, making it a popular choice for simulating various systems, particularly biomolecular systems. In the Martini model, groups of atoms are represented as single interaction sites, significantly reducing computational cost while retaining the essential chemical and physical properties of the system. In its original formulation, it is based on a four-to-one mapping, where four heavy atoms are grouped into one coarse-grained bead. One of the key benefits of the Martini model is its transferability as it has been parameterized to apply to a broad range of molecules, including polymers, lipids, proteins, and carbohydrates.^42−44^ This allows for the study of diverse systems without the need for extensive reparameterization.

The Martini model also balances simplicity with accuracy, by typically representing four heavy atoms with one CG bead;^43^ thus substantially reducing computational costs while maintaining a reasonably accurate representation of molecular interactions and no loss of accuracy in macroscopic property predictions. The model is also compatible with widely used MD software packages, such as GROMACS,^45^ further facilitating its adoption by the research community. The Martini model has been successfully employed to investigate various phenomena, such as membrane protein folding, self-assembly, and lipid–protein interactions, demonstrating its versatility and potential to provide insights into complex biological processes.^46,47^ Economou and co-workers employed the Martini CG force field to study *n*-alkanes,^48^ wax–water mixtures in Fischer–Tropsch synthesis,^49^ and catalyst nanoparticle behavior.^50^ Additionally, they compared its efficacy against a new methodology for amorphous amylose,^51^ highlighting Martini’s versatility in MD simulations across diverse applications.

CG models are widely used in the simulation of polymers due to their computational efficiency and ability to capture the essential features of polymer behavior. However, some limitations are also associated with the use of the CG models. First, the coarse-graining procedure can lead to the loss of some details and information at the atomistic level, which can affect the accuracy of the simulations.^52^ Second, selecting the proper CG scheme and parameterization can be challenging and require significant empirical testing and validation.^53^ Third, CG models may not be able to capture the subtle effects of external factors, such as solvent quality and confinement, on the behavior of polymers. Finally, the interpretation of CG simulation results can be complicated due to simplification of the system complexity, which can hinder the understanding of the underlying physics. Despite these limitations, CG models remain a valuable tool in the study of polymers, particularly for exploring large-scale phenomena that are difficult to study using atomistic models.

The use of Martini-based models for PIs has a set of several advantages over atomistic simulations: (1) the models are used to simulate interactions at the mesoscale level for nanoparticles and peptide systems,^50,54^ supporting the advantage of using Martini-based models to study polymer–filler interactions in mesoscale systems such as MMM. The Martini model can be used to study the properties of polymers at interfaces and confined geometries, such as in nanopores and on surfaces. (2) The models provide a path toward polymer screening and finding potential new applications due to the model’s transferability across functional groups.^55^ The Martini model can be used to study the interactions between polymers and solvents,^56^ including the solubility of small molecules in a polymer and the diffusion of small molecules through a polymer membrane. (3) The Martini model can be used to study the mechanical properties of polymers, including their elastic moduli and failure mechanisms, which are important for various applications including membrane science.

In this study, we developed CG force field parameters for a series of 6FDA polyimide polymers, utilizing Martini 3 bead types. Through an extrapolation strategy based on bead interaction tables, our methodology uniquely expands the nonbonded parameters of Martini 3 to accommodate bigger sizes of beads. This methodology demonstrated a high level of accuracy in replicating the structural and physicochemical properties of the polymers. We also investigated the important influence of the comonomer architecture on the characteristics of the polymers. Our work is distinguished by the transferability of proposed parameters across various polymers and by its notable computational efficiency. We performed gas separation simulations, including solubility and diffusion estimates for the 6FDA-DAM polymer, for some small adsorbates by utilizing these specially designed polymer force fields. Utilizing hybrid coarse-grained-atomistic (CG-AA) methods, these simulations achieved reasonable agreement with experimental data. Additionally, we propose statistical models that examine the variation in the force field parameters among polymers. With the use of this statistical approach, essential parameters including density, cohesive energy density (CED), glass transition temperature (*T*_g_), and mechanical properties may be predicted using basic atomistic descriptors such as molecular surface area and the distance between imide and diamine beads.

## 2 Methodology

In a CG simulation of a polymer, the first step is typically to create a CG representation of the macromolecular chain, which involves grouping atoms into beads and defining the potential energy function. There are many different methods for creating CG models, such as the united atom and the segmental bead models.^57,58^

**Figure 1:**
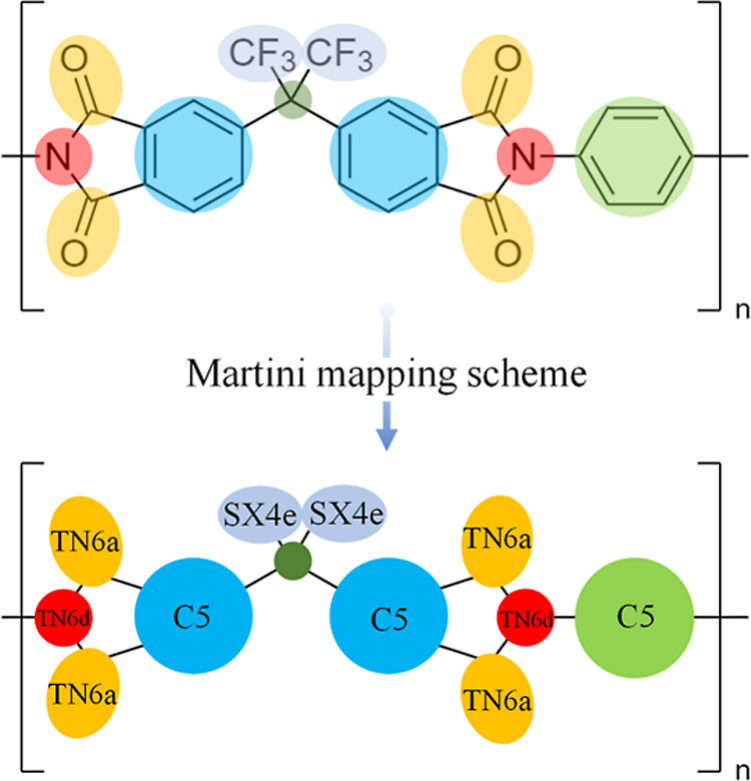
Mapping scheme used in the coarse grain modeling framework for the 6FDA-PPD building unit. The beads are labeled using the Martini 3 bead types.

In this work, suitable 2-to-1, 4-to-1, and 6-to-1 mappings were introduced as part of the CG modeling strategy for PIs. The beads used were assigned from Martini 3 bead types,^55^ with minimal alteration to achieve polymer symmetry. A representative example of the mapping scheme of the 6FDA-PPD polymer is shown in Figure 1. Clearly, the present model decreases the number of interaction sites for a 6FDA-PPD monomer from 39 to 12 beads (a 69% reduction). As a result, the CG representation improves computing performance by approximately 1 order of magnitude without loss of accuracy and allows simulation of phenomena with longer characteristic times.

In the present study, we employed the Martini 3 force field to model the nonbonded interactions in our CG macromolecular system. The Martini 3 force field is a widely used model in CG molecular simulation, designed to strike a balance between simplicity and accuracy for the representation of biomolecular and soft-matter systems, in general.^46,55^

**Figure 2:**
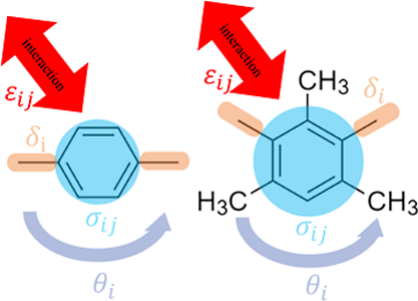
Demonstration of the four different parameters (σ_*ij*_, ε_*ij*_, δ_*i*_, and θ_*i*_) used in the force field parameterization for 6FDA-PPD (left) and 6FDA-DAM (right).

In this model, four different parameters are adjusted to characterize a diamine, as can be seen schematically in Figure 2. Two are bonded parameters: the catenation angle, θ_*i*_, and the bond length, δ_*i*_. Both significantly influence the PI density, radius of gyration, and glass transition temperature (*T*_g_). The catenation angle, or the angle between successive bonds in the polymer backbone, affects the polymer chain’s overall conformation and packing. The other two parameters (ε_*ij*_ and σ_*ij*_) are related to nonbonded interactions between polymer beads calculated by the Lennard-Jones (LJ) potential. In this work, we used the 12–6 form of the Lennard-Jones potential (eq S4 in the Supporting Information), and the parameters are provided in the Supporting Information.

**Figure 3:**
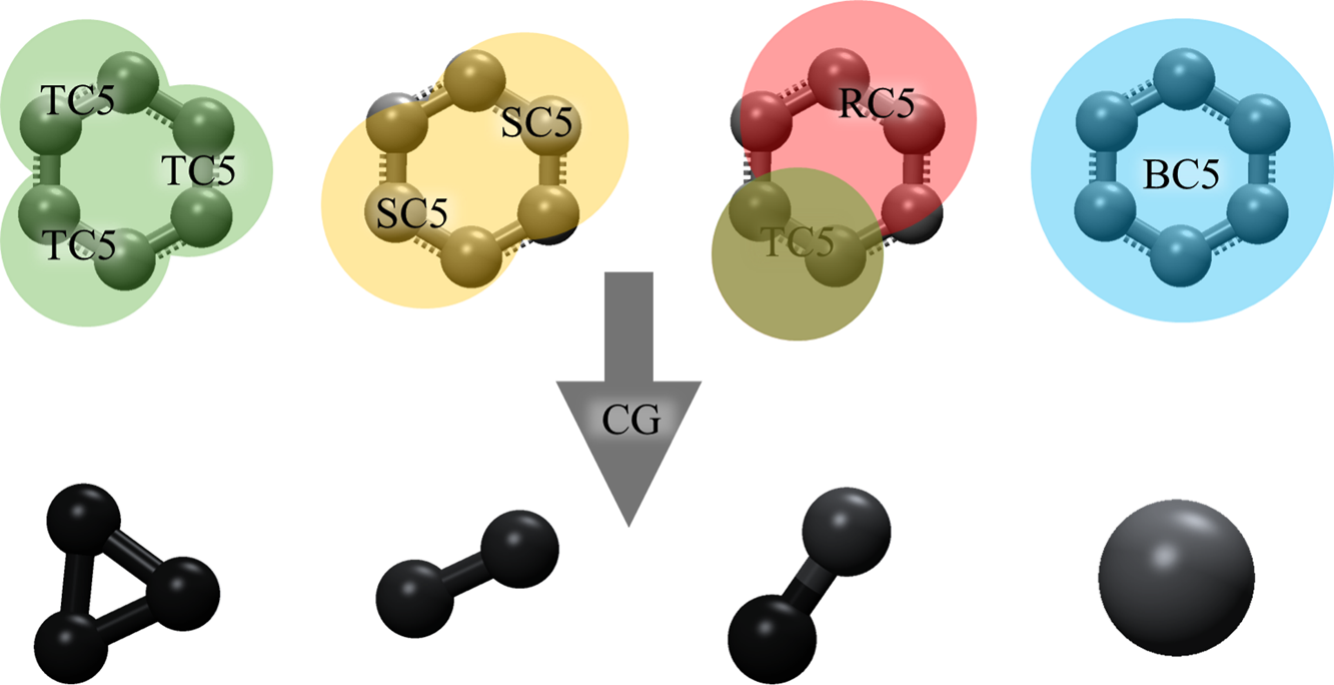
Different coarse-grained representations of the benzene ring using Martini 3 with tiny (T), small (S), and regular (R) beads. C5 type of bead is used to represent dienes in Martini 3. The figure shows the bead BC5 used in this work for modeling benzene ring in 6FDA monomer and PPD and MPD diamine monomer beads.

Creation of new beads depends on the different levels of mapping and the type of atomic grouping. The fabrication of modified beads is contingent upon varying mapping degrees and the specific atomic grouping. While the literature has yet to present the expansion to larger Martini 3 beads (incorporating more than 4 heavy atoms into a single bead), it is important to acknowledge that beads comprising a higher number of atoms (>4) have been documented in studies examining anions in ionic liquids.^59^ This extension is critical to screen for various PIs and glassy polymers quickly. In the current work, we used two new beads, BC5 and G, representing 6 to 1 mapping of the benzene ring in 6FDA monomer, and the diamine comonomer mapped to a single bead, respectively. The size of bead BC5 is fixed at 5.4 Å, as shown in Figure 3, while the size of bead G varies and is determined by the size of the diamine in the corresponding polymer.

The nonbonded interactions are modeled from the established extended interaction table. The LJ parameters for the carbon connecting the two CF3 groups, shown as dark green in Figure 1, in 6FDA monomer are transferred from atomistic simulations, and parameters are obtained from PCFF.^60^ To build the nonbonded interactions in our system, we first assigned appropriate Martini 3 particle types to each CG bead, following the guidelines provided by the developers of the force field.^55^ Interactions are defined per Martini 3, and the newly added beads are established by extending the tables of interactions to larger-size beads by extrapolation/interpolation of data. The size of the diamine is determined from the molecular surface area of the atomistic structure using defined van der Waals radii of the atoms comprising the bead using eq S6 (refer to Section S2 in the Supporting Information for nonbonded parameters estimation). Bonded interactions and force constants were extracted from the work of Pandiyan et al. for eight-bead CG models with modifications depending on the diamine comonomer used.^26^ The catenation angles and bonds are adjusted per the moiety of considerations based on atomistic simulation and geometrical optimization of the trimer polymeric chain, using PCFF, by leveraging the center of the beads for estimations of bonds and angles. A full list of CG force field parameters utilized in this work are presented in the Supporting Information document and force field functional forms are reported in eqs S1–S5.

The polymer beads are assumed to be chargeless since the partial charge of each bead was less than 0.25*e*. After defining the nonbonded interactions, we carried out simulations using GROMACS, a widely employed MD simulation package.^45^ A cutoff of 11 Å was applied for the van der Waals interactions. The integration time step was set to 2 fs, consistent with the bonded interactions. 20 ns long *NVE* MD simulations were performed to monitor the energy conservation at 1 and 2 fs time steps. The root-mean-square (RMS) fluctuations in the total energy were 0.008% and 0.01% and in the potential energy were 0.075% and 0.078%, respectively.

### 2.1 Optimization of the Polymeric Structure

In this work, the initial configurations were prepared by inserting 10 trimer chains using Monte Carlo (MC) steps involving a regrowth algorithm (a representation of the initial box is shown in Figure S4). The use of trimer chains in our initial simulations was primarily motivated by computational efficiency during the validation phase and to test the transferability across different polymers studied in this work. However, we acknowledge the importance of longer chains and system size in order to capture accurately the polymer properties, as highlighted in previous atomistic model simulations.^61^ As an example, Pandiyan et al.^61^ used chains with 50 monomers and reported that chains longer than 25 monomers showed a plateau in the normalized mean-square end-to-end distance, indicating that longer chains are necessary to observe the characteristic scaling behavior of polymers.

To systematically investigate the effect of the degree of polymerization on density and to address potential finite-size effects, we performed simulations with larger system sizes. Specifically, we simulated 6FDA-DAM polymers with chain lengths of 5, 10, 20, 40, 50, 100, 200, and 400 monomers. In addition, to extend beyond the initial system of 10 trimers, we increased the system size up to 200 trimers, resulting in a total of 7200 beads and approximately 33,800 atoms. This system size surpasses those typically studied in atomistic simulations, which generally range between 8000 and 20,000 atoms,^61^ thereby ensuring a more representative bulk-like behavior. These simulations confirm that longer chains have a slightly higher density than the trimer but within the statistical uncertainty of the simulations, while the chain structural properties expressed by the ratio  remain unchanged. So, although the newly proposed parameters here can be used for longer molecular weights, experimental density values for similar polyimides with a broad molecular weight from 45 kDa (approximately 80 monomers) to more than 100 kDa (approximately 178 monomers) show a clear molecular weight effect.^62,63^

The annealing process was adopted to ensure local energy minimization of the glassy polymer. The simulations were initiated using a cubic box, as previously mentioned; however, the simulations were conducted under triclinic box conditions, allowing all cell parameters, including cell lengths and angles, to equilibrate dynamically in the *NPT* ensemble. The process starts with energy minimization, followed by *NVT* thermalization at 300 K for 1 ns. After that, an *NPT* heating (from 300 to 800 K) is accomplished at a rate of 250 K/ns. The process of cooling used is a slow process that includes intermittent *NPT* simulations. Cooling was done at 25 K/ns, followed by *NPT* equilibration at the reached temperature to estimate the density and equilibration of the structure. This was followed until the temperature reached 300 K. The effect of the cooling rate and annealing cycle on the polymer structure was also examined in this study. Three annealing cycles were used throughout based on optimization of the number of cycles that provide consistent polymer chain structure properties (radius of gyration and end-to-end distance) of 6FDA-DAM. During the annealing process, temperature control was achieved using the V-rescale thermostat with a time constant (τ_t_) of 0.1 ps. This thermostat was able to maintain accurate temperature control with minimal fluctuations. Pressure was controlled using a Parrinello–Rahman barostat, with pressure coupling and a time constant (τ_p_) of 14 ps. This setup was implemented to ensure pressure conditions, which are essential for the stability of the simulation system during the annealing process. Following the annealing process, *NPT* ensemble equilibration was accomplished to determine density at a given temperature. In these calculations, Berendsen thermostat and barostat (with pressure coupling) were used with time constants of 0.1 and 12 ps, respectively.

For *T*_g_ calculations, we heated the polymer structure to 1000 K, followed by cooling it in 25 K increments. The cooling was performed at various rates to examine the influence of the cooling rate on *T*_g_. After each cooling step, a 5−10 ns equilibration period was used to stabilize the system at the target temperature, using a Berendsen thermostat and barostat with time constants of 0.1 and 12 ps, respectively, during which the molar volume was recorded.

### 2.2 Simulation Details for Sorption Calculations

Equilibrium adsorption isotherms at 308 K in the 6FDA-DAM polymer were computed with grand canonical Monte Carlo (GCMC) simulation using the Cassandra code.^64^ In the GCMC, the system temperature, volume, and chemical potential are fixed. The chemical potential values of guest molecules were obtained by independent bulk gas simulations, to correlate input chemical potential to fugacity, and used subsequently as input in the GCMC simulations. The TraPPE force field was used to model CO_2_, N_2_, O_2_, CH_4_, propylene, and propane.^65−67^ TraPPE force field parameterization is based on reproducing vapor–liquid equilibria from low temperature up to close to the critical point and on molecular structure. The TraPPE force field has been extensively utilized in various applications including polymer systems, particularly for simulating gas transport properties in membranes, effectively predicting CO_2_ solubility, diffusivity, and permeability, as highlighted by multitask learning approaches and high-throughput computational simulations.^68,69^ TraPPE force field parameters are listed in Tables S2 and S3. For a given gas at a specific temperature, gas phase GCMC simulations were carried out to develop a relationship between the chemical potential (μ) and fugacity of the gas (Figure S2). The Martini force field was previously shown to perform adequately in a hybrid simulation of atomistic peptides in CG butane and water.^70^

The absorbates in this study are non-polar or only weakly polar; polar molecules such as water would require explicit electrostatic coupling of the AA/CG system.^71^ Hybrid AA/CG simulation is needed for evaluating gas separation performance as it captures the effect of molecular details of the adsorbate on diffusivity and adsorption. The approach is particularly relevant in this work for polymers with relatively small channels and pockets. Adsorbate moves include translation, rotation, insertion, and deletion. The trial insertions of penetrant molecules were performed randomly throughout the polymer matrix without the use of any bias method.

The polymer was modeled by using the initial conformation from the annealing MD simulation. Cycles of GCMC and MD simulations were implemented to investigate sorption-induced volume swelling and plasticization. These cycles involved alternating sorption and relaxation steps to achieve an equilibrium sorption amount. In each cycle, the polymer matrix was first loaded with sorbate molecules during the GCMC step. The loaded matrix was then subjected to an *NPT* (constant number of particles, pressure, and temperature) simulation for 5–10 ns, allowing the system to reach an optimized and equilibrated state. After the *NPT* simulation, the sorbates were removed from the matrix and the matrix was prepared for the next GCMC step to determine its equilibrium sorption capacity.

We used configurational-bias MC (CBMC) moves to allow the polymeric chains to reconfigure and swell in the presence of small molecules (the distribution of moves is reported in Table S26). The use of CBMC for the polymer accounts for changes in polymer flexibility and penetrant interactions, which are important in glassy polymeric systems. As discussed in the literature, advanced techniques such as configurational-bias and expanded ensemble methods are necessary to overcome these challenges and model solubility accurately.^72^ In GCMC simulations, we allow the number of particles in the system to vary. This approach is particularly useful when one wants to examine scenarios where particles are exchanged with a reservoir such as when gas is adsorbed into a porous material. Using these simulations, one can calculate the quantity of gas that gets adsorbed under specific chemical potential and temperature conditions. This data gives us valuable insights into the material’s adsorption capacity and affinity toward the gas in question.

Lorentz–Berthelot mixing rules were used to describe adsorbate–polymer interactions, while the Lorentz rule was modified according to eq S5 to include a softness parameter as per Martini 3 implementation. The van der Waals interactions were cut off at 11 Å. The Ewald summation was used to compute long-range Coulombic interactions for the systems where such interactions exist, as in the case of atomistic simulations and when CG with bead charges were used. Tabulated energy and Coulombic interaction grids were generated with a 0.25 Å spacing to enhance the computational efficiency. The adsorption simulations were performed with 1 × 10^6^ cycles of initialization and 2 × 10^8^ cycles for production.

## 3 Results and Discussion

### 3.1 Molecular Simulation: Physical and Structural Properties

Table 1 provides a comprehensive list of various diamines employed in the construction of PIs in this study. For each diamine, we provide the structural attributes, specifically the molecular surface area and the size of the bead. The molecular surface area of each diamine is used as the primary parameter to understand the space occupied by the diamine. The surface area of the atomistic structure is determined using the well-defined van der Waals radii of the atoms of the bead. Correspondingly, the bead size of each diamine is also presented. The table also lists the catenation angle value used to represent the angle connecting the imide group and the diamine bead. The types of Martini 3 beads used to model diamines are listed in Table 1. The types of beads and the corresponding parameters utilized for the 6FDA monomer are listed in Table S4.

**Table 1:**
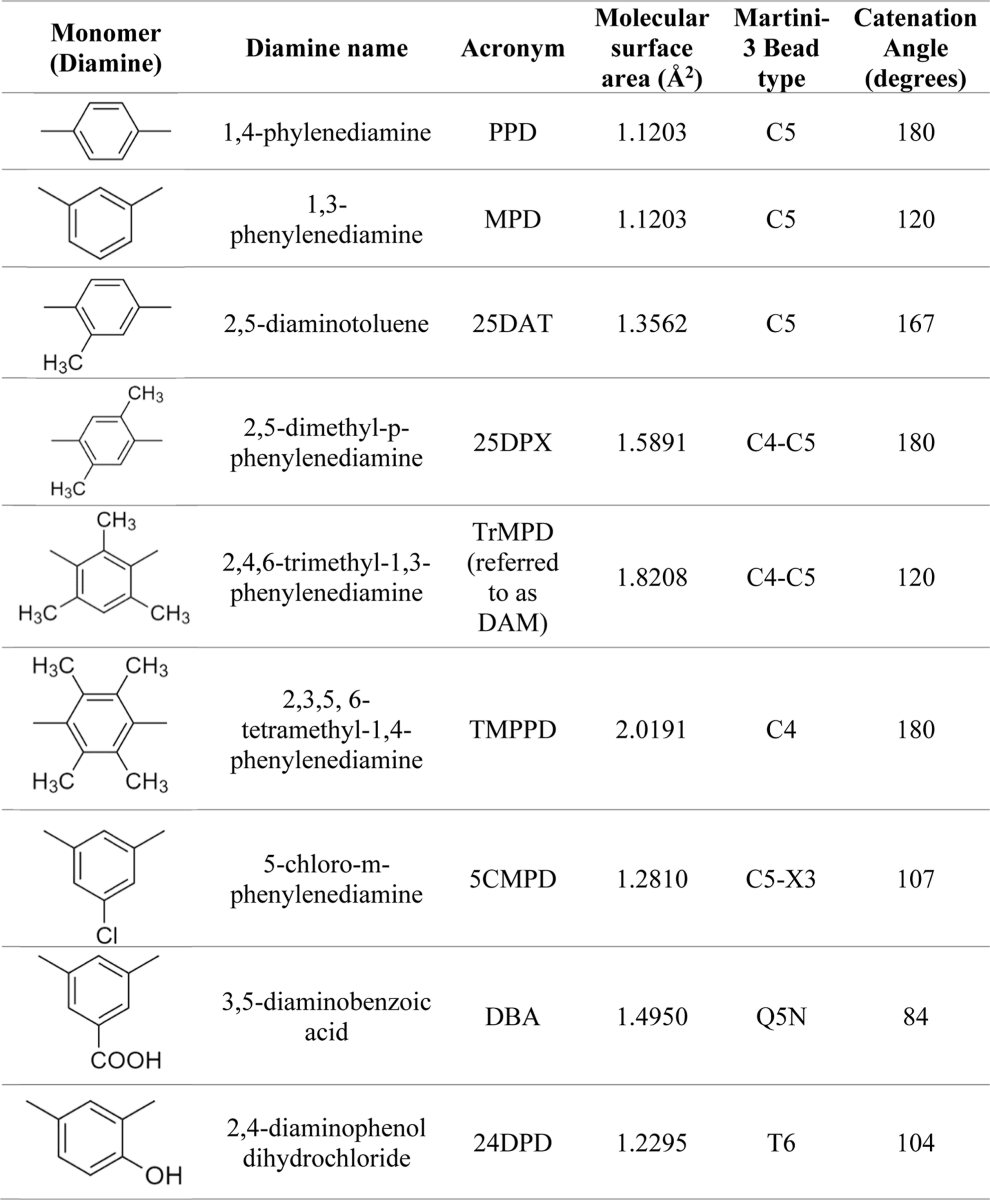
Type of Monomers, Corresponding Molecular Surface Area, and the Catenation Angle in Each Monomer Used in Martini 3

**Figure 4:**
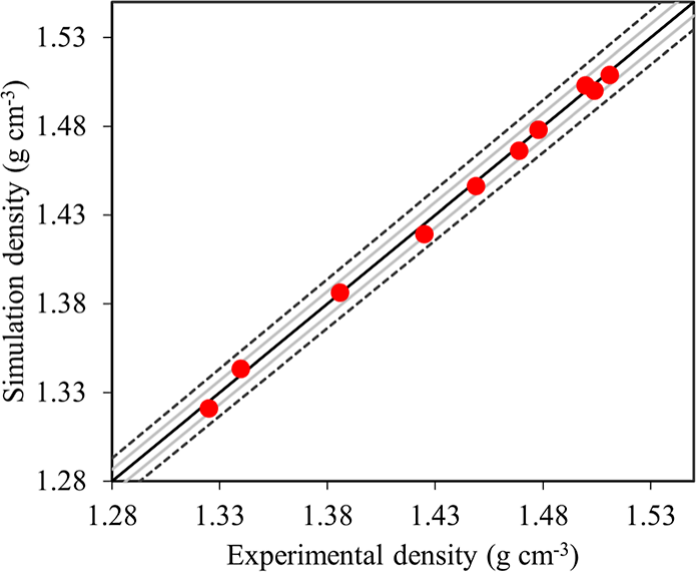
Calculated mass density of various polymers compared to experimentally measured value at 298 K.^73^ The continuous light dark gray and dashed lines show 0.5% and 1% relative deviation, respectively. The density calculations were used to evaluate the transferability of the model across different diamine structures.

**Figure 5:**
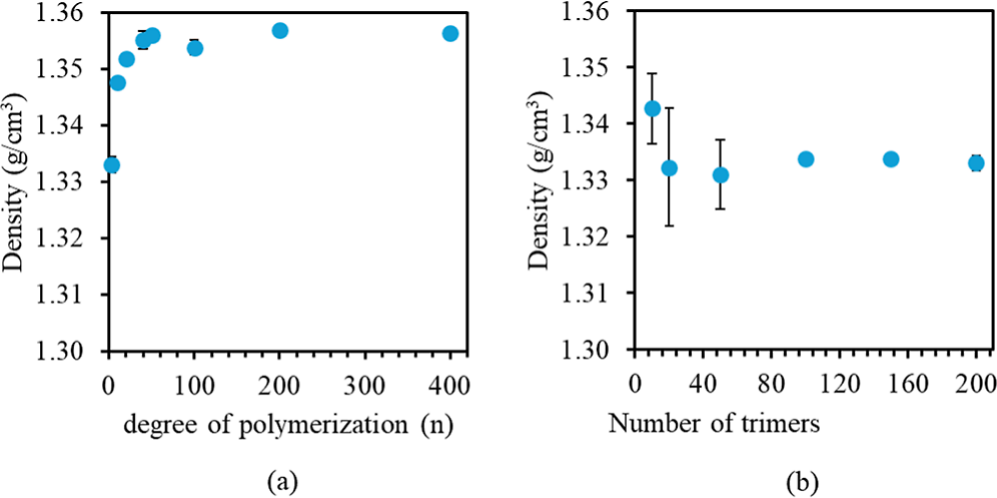
Calculated mass density of the 6FDA-DAM polymer at 298 K as a function of (a) chain length (degree of polymerization) of up to 400 monomers and (b) system size of up to 200 polymer trimers.

In Figure 4, a comparison of the experimental and predicted densities at 298 K for the series of studied polymers is presented. This comparison is very important as it validates the accuracy of our simulation model in predicting a key physical property. The ability to accurately predict the density and, consequently, the specific volume is of utmost importance. Here, we show that CG simulation is a computationally efficient method to predict the density by modifying a few parameters in the force field. Most of the simulation densities are within 0.5% of the experimental values. Figure 4 also shows that a small catenation angle results in a more compact and denser structure, leading to increased density. This is directly illustrated by the density increase from MPD to PPD diamines. These two polymers have precisely the same force field parameters, except for the catenation angle. It is worth mentioning that the density is estimated after three annealing cycles, as discussed in the Methodology. In Figure S5, details are provided to justify the use of 3 annealing steps. The results in Figure 4 demonstrate good agreement between the experimental data and simulations. To further investigate the impact of molecular weight on polymer density, we conducted additional simulations with extended chain lengths of up to 400 6FDA-DAM monomers (Figure 5a). The results indicate that polymer density initially increases with chain length due to the diminishing influence of chain-end free volume effects before stabilizing and reaching a plateau at approximately 20 monomers, in agreement with experimental behavior.^74^ To ensure that these observations could be solely attributable to the degree of polymerization rather than system size artifacts, we maintained a consistent total number of beads across all simulations. The reported experimental density of 6FDA-DAM in the literature ranges from 1.259 g/cm^3^ to 1.37 g/cm^3^, with most studies reporting a value of 1.35 g/cm^3^.^62,63,73,75−81^ The effect of the system size is shown in Figure 5b as the number of polymer chains (trimers) increases. The results reflect the average density of three independent initial structures with a starting density of 1.3 g/cm^3^.

In this work, we also studied the effect of introducing charges to the PI, and to each bead, the summation of the partial charges of atoms included in the bead was assigned. In a case study, charges introduced in CG of 6FDA-DAM based on the charge equilibration method of Gasteiger^82^ affect the density of the polymer by about 1% (charges used for 6FDA-DAM are available in Table S18). This behavior also holds with other polar groups such as chlorine (5CMPD) and carboxylic groups (DBA) because of the protocol used to calculate the charges of beads by combining partial charges on atoms comprising the specific bead. From this, we highlight one of the shortages in using CG modeling in these cases. It has been reported in the literature that the electrostatic interactions in atomistic simulations are responsible for some changes in the thermophysical properties of PI upon modification of their chemical structure by introduction of the polar groups. In fact, changes in flexibility and specific volume have a weaker effect on the thermophysical properties. Due to strong electrostatic interactions between polar groups, some local structural ordering of PI fragments occurs.^83^

**Figure 6:**
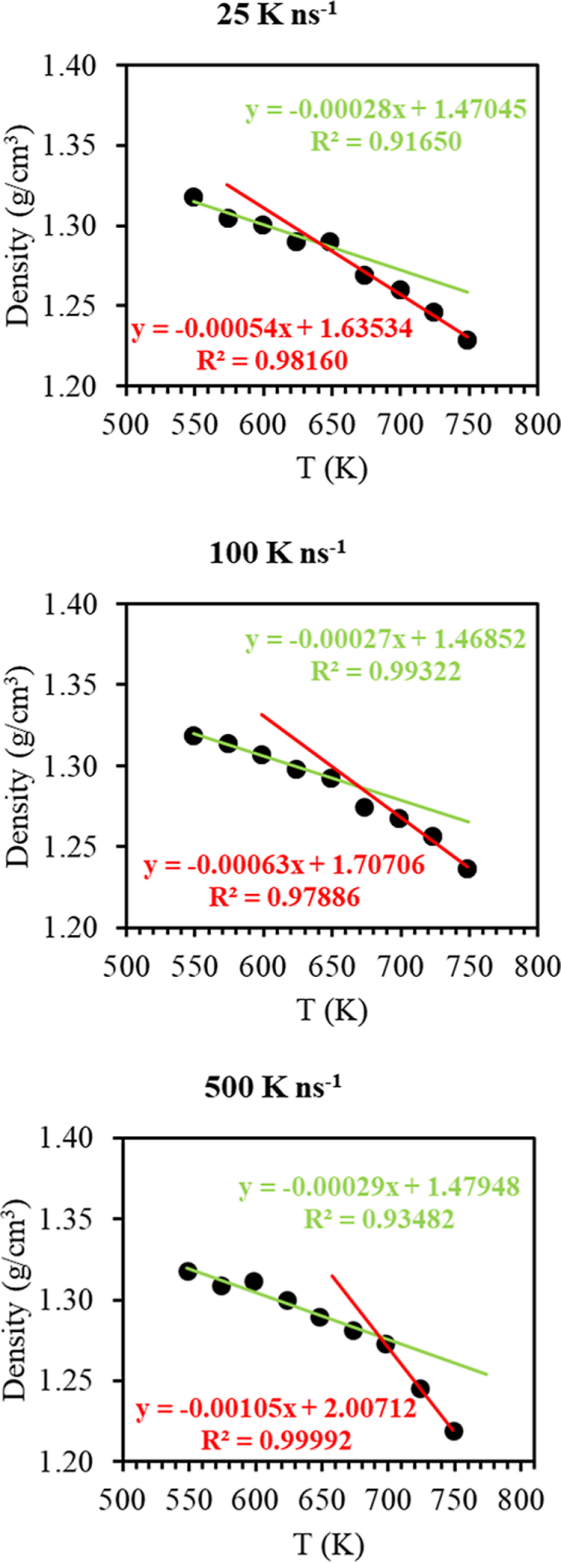
Density of 6FDA-DAM PI calculated from MD simulations as a function of temperature at 1 bar and at three different cooling rates. The crossover point of the two lines represents the estimated *T*_g_ in each plot. Error bars are smaller than the symbols.

To study the effect of temperature on polymer properties, we simulated the PIs between 500 and 800 K (temperature step of 25 K), and their *T*_g_ values were determined. In Figure 6, the density of 6FDA-DAM at 1 bar is shown as a function of the temperature. The initial structures used for *NpT* simulations were extracted from the third cycle of the annealing process, including various slow cooling steps. A widely used approach was implemented,^84^ according to which, density values at low and at high temperatures are fitted to straight lines and the intersection of the two lines in each case provides the corresponding *T*_g_. The intersection point of the two straight lines drawn through a least-squares fitting to the individual simulation data predicts a *T*_g_ value of approximately 660–700 K, depending on the cooling rate implemented shown in Figure 6, which compares excellently with available experimental data (640–670 K).^61,73,77,79^ This shows an interesting result that emphasizes the importance of the CG model, allowing for relatively faster simulations to include slow cooling steps. Also, this result confirms the excellent accuracy of the polymer force field over a broad temperature range.

We extended the force field development for halogenated diamines by introducing F, Br, and I substituents into the phenylene linkage. We investigated the impact of substituting chlorine with fluorine, bromine, or iodine (in 5-chloro-*m*-phenylenediamine) on the density, and the results are shown in Table 2. This has been possible since we illustrated the potential transferability of the force field parameters by using atomistic variables and indicators. Also, Martini 3 beads include parameterization for halogenations, which is as follows: F, Br, and I are indicated with X4, X2, and X1 bead types, respectively, following the implementation of Martini 3. Interactions energies are similar between organic beads (C4, C5) and X(1–4) beads; however, F(X1) has the highest interactions with N6 beads, which are used to model the carbonyl group and nitrogen sites on the imide. Results of specific volume difference between F and I halogenated phenylene showed agreement with atomistic simulation using the PCFF force field.^60^ Given the specific volume and shape of the polymer, the free volume can be adjusted to target specific separations.

**Table 2:**
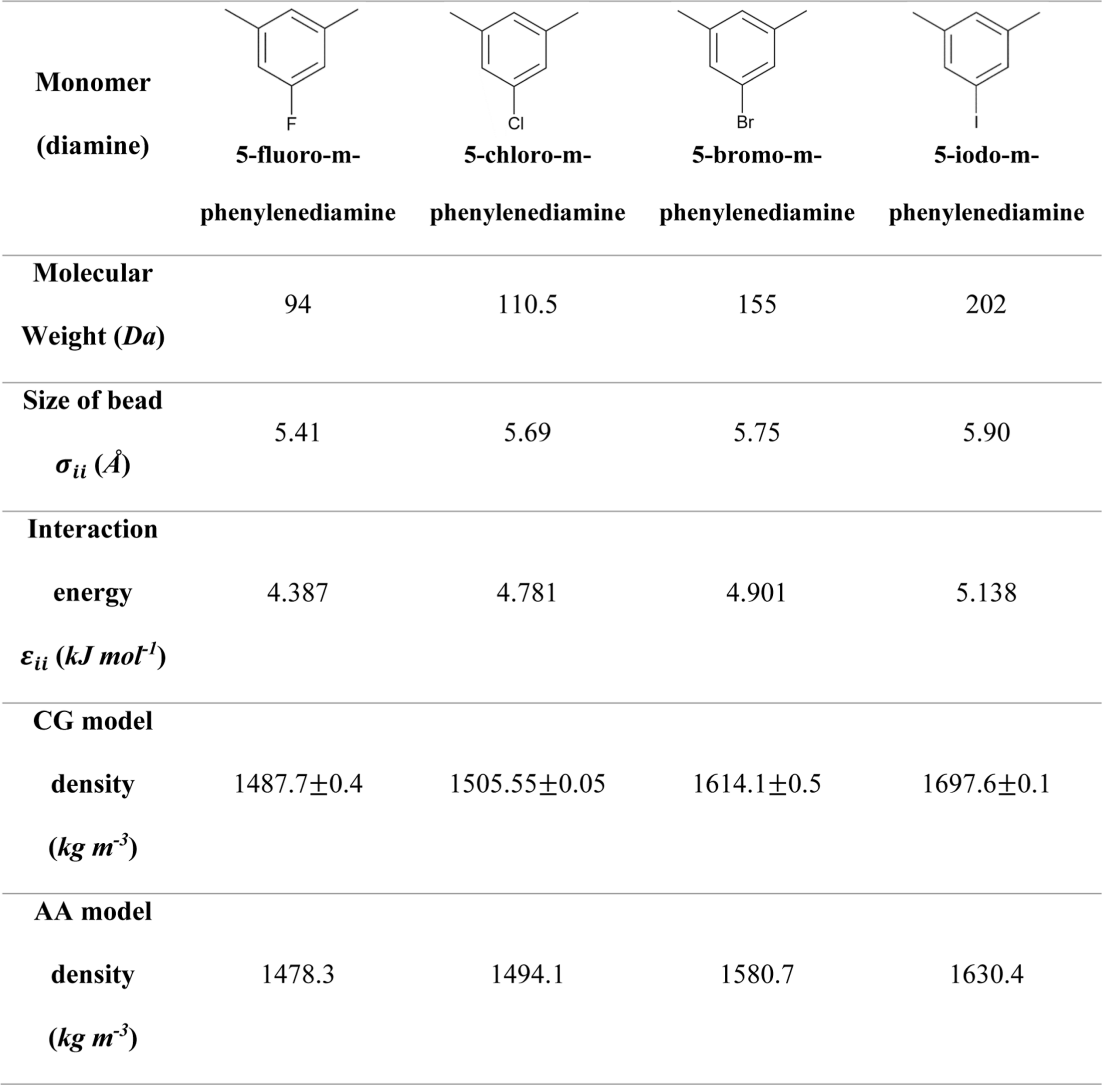
Influence of Halogen Group Replacement on Nonbonded Interaction Parameters and Predicted Density

Our results indicate that the polymer properties are significantly influenced by halogen substitution. When replacing chlorine with fluorine, we observed a decrease in density, which can be attributed to the smaller atomic radius of fluorine, leading to a more compact polymer structure.^85^ Conversely, when substituting chlorine with bromine and iodine, we noted an increase in density and a decrease in free volume owing to their larger atomic radii and enhanced van der Waals interactions. These findings are consistent with previous research on halogenated PIs,^86^ highlighting the importance of halogen substitution in tuning the properties of these high-performance materials.

**Figure 7:**
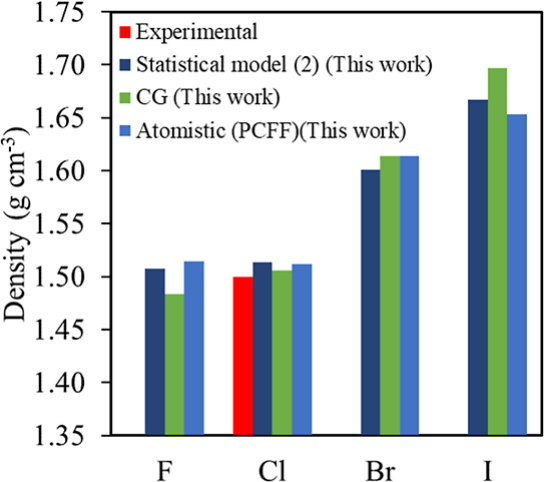
Density values for halogenated phenylenediamines: Experimental data, predictions from atomistic and CG simulations and from statistical model 2. Statistical model parameters are shown in Table S21 and refer to eq S7. The error bars in density calculations are smaller than 0.01 g cm^–3^.

In this work, we performed statistical modeling of polymer properties such as density, *T*_g_, and cohesive energy density (CED). CED is a measure of the total energy of the polymer per unit volume, which is evaluated as the sum of the intermolecular forces; thus, a higher value translates to stronger interchain interactions. One of the major goals is to establish design principles using information gathered from molecular simulation. Multiple linear regression models are presented using different parameters to model the different polymers, which were fitted to the bulk density, *T*_g_, and CED values of the polymers studied. A detailed description of the methodology used in the statistical modeling can be found in Supporting Information. The density predictive models have been tested to predict the mass density of halogenated diamine polyimides, and the results are presented in Figure 7. Two potential models were compared to the CG, atomistic (based on PCFF,^60^ atom types and nonbonded interactions shown in Figure S7 and Table S20) extension to halogenated diamines (Table 2). Model 2, for which parameters are shown in Table S21, includes MW_monomer_, which is important to predict the heavy atom effect such as Br and I on density. The model also includes terms related to size (σ) and the comonomer distance (*l*). It can be observed that this model agrees with atomistic simulations as the error relative to them is less than 1%, on average. This agreement justifies the importance of adding the molecular weight of the monomer term. One potential way to test the physical meaning of the statistical models is to understand what happens if there is no diamine in the system, essentially referring to the 6FDA monomer. In this case, statistical model 2 predicts a density of 1.88 g cm^–3^, which in principle corresponds to the density of 6FDA monomer (6FDA experimental mass density is about 1.7 ± 0.1 g cm^–3^).

While our model demonstrated excellent performance in predicting the density of polymers, it is important to note its satisfactory accuracy for other parameters such as free volume, *T*_g_, and CED. Figure S9 and Tables S22–S24 in the Supporting Information show prediction of *T*_g_ and CED versus experimentally reported values, for the polymers listed in Table 1.

**Figure 8:**
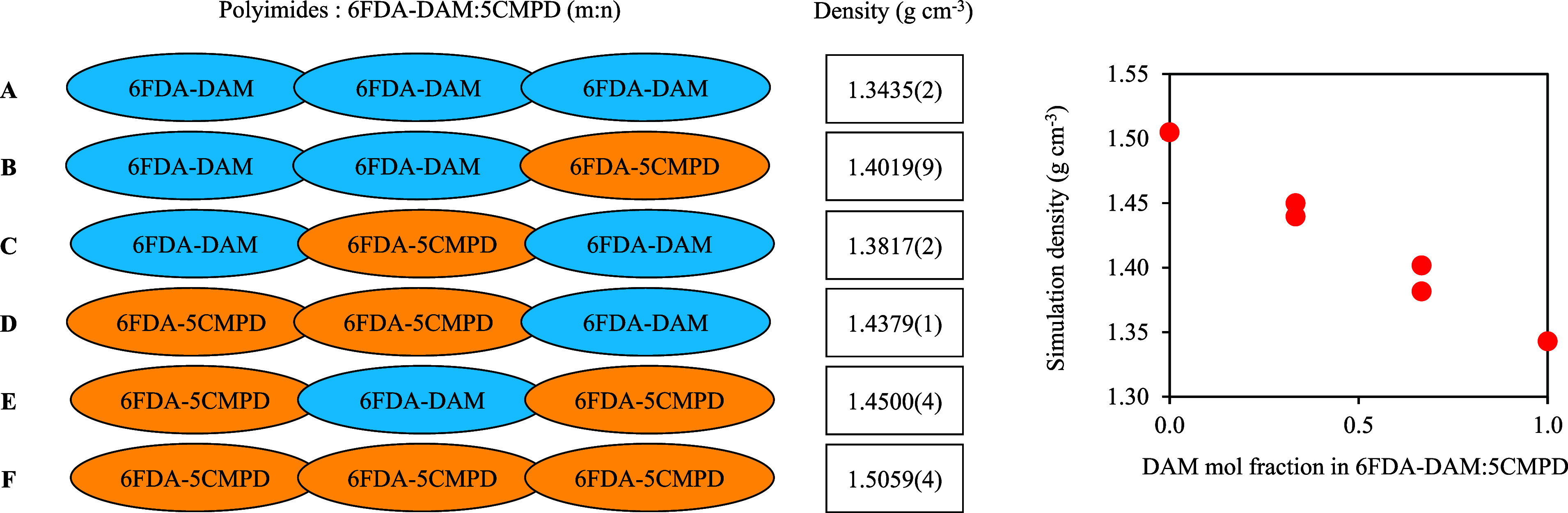
Molecular architecture and mass density from CG simulations of 6FDA-DAM/5CMPD (m/n) as a function of diamine mole fraction.

One of the methods to tune the thermodynamic and separation properties of the PI is by adjusting the ratio of the different diamines used in a copolymer. 6FDA-DAM/DABA (3:2) and 6FDA-DAM/mPDA (3:2) have been synthesized and tested for specified gas separation performance.^80^ Here, we demonstrate the use of CG models in predicting the thermodynamic properties of such copolymers. The example used in this work refers to 6FDA-DAM/5CMPD (m/n), where we varied the composition and the architecture of the trimer used (see the schematic representation in Figure 8). As anticipated, the resulting polymer density calculated from *NpT* MD simulations using the newly proposed CG force field increases as a higher proportion of 5CMPD diamine is present. The observation aligns with the inherent characteristic of the pure 5CMPD polymer, which exhibits higher packing than DAM. The trend highlights the opportunity to control some polymer properties by adjusting the diamine ratios involved.

Interestingly, Figure 8 also reveals that the order of diamines in the polymer chain can influence the density as well. Specifically, higher densities are achieved when 5CMPD diamine is positioned at the end points of the polymer. The phenomenon may be attributed to how the PIs arrange themselves and the subsequent intermolecular interactions. Although no specific literature is available to elucidate this observation further, it is possible that 5CMPD at the chain ends could promote a more compact coil structure, thereby leading to an increased density. In fact, our simulations show that the ratio of the mean squared radius of gyration to the mean squared end-to-end distance of DAM and 5CMPD are 6.12 and 4.73, respectively, at 300 K and 1 bar, which support the claim mentioned above. This finding emphasizes the role of diamine order and composition in determining polymer properties. Moreover, it suggests that careful considerations of both the diamine ratio and order as such interactions could be favorable to control the separation and interfacial energy with fillers in MMM.

Structural properties are important as they have a role in determining polymers’ efficiency toward gas separation performance. First, we tested the CG model and compared it with atomistic simulations in terms of the radial distribution function (RDF). The calculations for H and E beads are reported in Figure S10. Next, we studied the radius of gyration, end-to-end distance of chains, accessible area, and pore size distribution (PSD) of the prototype trimers. PSD histograms were calculated using the zeo++ package;^87^ they indicate the fraction of the void space volume that corresponds to certain pore sizes. PSD is calculated using an MC approach similar to the determination of accessible volume calculations at a given pore size.

Table S25 lists the polymers along with their simulated radius of gyration, end-to-end distance, and the ratio of the mean squared radius of gyration to the mean squared end-to-end distance after the third annealing step for the trimer system. This dimensionless ratio provides valuable insight into the conformational properties of the polymer chains in the melt. The polymers in the table exhibit a range of values, suggesting a variety of chain conformations. Most polymers exhibit ratios between 5.8 and 6.4, respectively, closely aligned with the ideal chain model.^88^Figure S5 discussed previously in the manuscript demonstrated the effect of various cooling rates cycles on the structure of the 6FDA-DAM system.

To test the accuracy of the new force field for longer chains, we simulated a 20-mer 6FDA-DAM polymer. The calculated radius of gyration was approximately 28 Å, which compares well with atomistic simulations [∼(31 ± 5) Å].^89^ This finding is an additional indication of the transferability of the CG model developed here to longer chains. In addition, the effect of the system size on the conformation ratio can be observed in Figure S11.

The peak of the PSD curve represents the most common or dominant pore size. This can be important for applications that require a specific pore size, such as filtration or separation processes. For example, in gas separation, the dominant pore size can determine which gases can permeate the material and at what rate. From the structures studied here, the peak of the PSD curves of PPD, 5CMPD, TMPPD, and DAM is at 4.8 5.0, 5.2, and 5.3 Å, as shown in Figure S12a, respectively. The width of the PSD curve indicates the range of pore sizes. For example, a broad pore size distribution might enhance the material adsorption capacity for various molecules but could reduce its selectivity in a separation process. DAM and TMPDD PIs have pores larger than 7 Å, which may highlight improved permeability relative to the other diamines reported in experimental findings.^73^Figure S12b shows a direct comparison of PSD between atomistic and CG models for the 6FDA-DAM polymer. The figure suggests that CG models show larger cavities, as seen in the distribution.

A further look at a snapshot of 6FDA-DAM was made by investigating the channels and pockets in the structure using a probe of size 0.45 Å that has been shown previously to provide the closest estimation of free volume to Bondi’s approach.^90^ The estimation of the free volume using the Bondi approach is closely linked to the accuracy of the predicted density. The calculated fractional free volume is approximately 0.193, which compares well with the experimental value of 0.189.^73^ The study showed that the structure has an accessible surface area of 1233.5 m^2^ g^–1^, and the single channel has an area of 3441.24 Å^2^. Within a volume of 21 nm^3^, the structure has 33 different pockets with a wide range of surface area, with a maximum of 17.2 Å^2^, which corresponds to 6.7 Å^3^, assuming a spherical pocket. In comparison to the atomistic trajectory, the values of the largest included spheres were 4.54 and 4.17 Å for the CG and AA, respectively.

We have calculated the self-diffusion coefficient of polymers at 500, 650, and 800 K to assess the polymer dynamics using the proposed force field, and results are shown in Figure S13. The diffusion coefficient value found at 650 K is of the order of [2–5] × 10^–8^ cm^2^/s and compares well with atomistic simulation calculations carried out for HFPE-30, a polyimide with a similar backbone (9.3 × 10^–8^ cm^2^/s at 650 K).^26^

### 3.2 Molecular Simulation Studies of Gas Separation and Mechanical Properties

**Figure 9:**
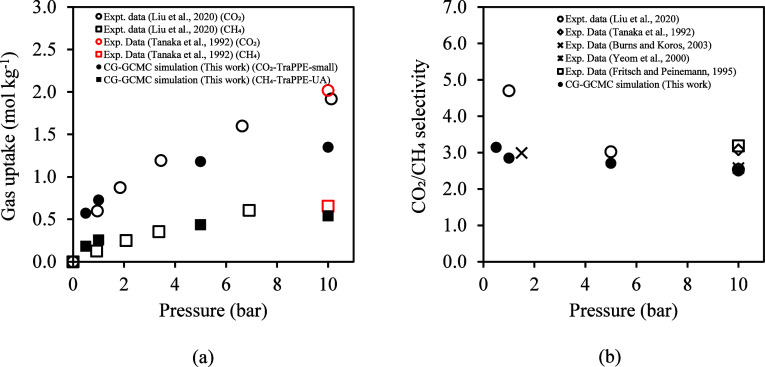
(a) Experimental solubility measurements^75,92^ and molecular simulations for CO_2_ and CH_4_ in 6FDA-DAM at 308 K considering only polymer flexibility. (b) Experimental data.^75,76,79,91,92^ and molecular simulation predictions for CO_2_/CH_4_ selectivity.

The major focus of this work is modeling the 6FDA-DAM polymer for specific applications. It has to be taken into consideration that the literature-reported experimental density of 6FDA-DAM at 298 K ranges from 1.259 g/cm^3^ to 1.37 g/cm^3^.^62,63,73,75−81^ Such a difference in density should highly affect the void distribution and fractional volume, which impacts the solubility and transport properties. For details regarding probability move allocation in GCMC, refer to Table S26 for moves used to optimize the convergence of the simulations. Figure 9a provides a comparison of the simulation sorption for CO_2_ and CH_4_ with experimental data. Overall, there is good agreement between experimental data and calculations; however, an underestimation of the CO_2_ uptake is observed at higher pressures. Two other experimental studies reported considerably higher CO_2_ and CH_4_ sorption (double and triple the uptake of CO_2_ at 10 bar, respectively^76,79^) which is not included in Figure 9a. In this work, GCMC simulation accounts for the flexibility and interaction effect of adsorbates, and GCMC/*NPT* cycles are included to account for the volume change as the adsorption occurs (Figure S14). In addition, we estimated the difference of including point charge on polymer beads, and the results show that absolute uptake of CO_2_, at 308 K and 10 bar, is reduced by approximately 15%. In Figure 9b, a good agreement between the calculated selectivities and experimental data at different pressures is shown.

In Table 3, the uptake and selectivity of O_2_, N_2_, propane, and propylene at conditions comparable to experimentally available data are shown. Results demonstrate an excellent agreement between calculated selectivities and experimental results. On the other hand, the amount of adsorbed propane and propylene is lower than that reported in the experimental studies. It is worth mentioning that long GCMC simulations were performed to ensure sufficient time for the polymer to adopt a new configuration as a result of the presence of adsorbed gases. To demonstrate the efficiency of incorporating CBMC thermal moves in sampling conformational changes of the glassy polymer, we plotted the average sorption of CO_2_ and propylene as a function of MC steps and compared the results with and without accounting for polymer flexibility (Figure S15). Figure S16 illustrates the relationship between the critical temperature of gases (CO_2_, CH_4_, N_2_, and O_2_) and their respective solubility. Leveraging this relationship, the propylene uptake at 1 bar and 308 K is anticipated to be 0.98 mol kg^–1^. This prediction aligns well with experimental findings, although the GCMC simulation for propylene indicates a solubility underestimation, as reflected in Table 3.

**Table 3:** 6FDA-DAM Solubility for O_2_, N_2_, Propane, Propylene, O_2_/N_2_, and Propylene/Propane Solubility Ratios and Comparison to Literature Experimental Data without Considering Swelling^a^

adsorbate	condition	solubility (mol kg^–1^) and solubility ratios	
		this work	exp values with refs
O_2_	308 K and 10 bar	0.430 ± 0.007	0.081,^75^ 1.260,^79^ 0.157^76^
N_2_		0.276 ± 0.004	0.066,^75^ 0.730,^79^ 0.101^76^
O_2_/N_2_		1.56 ± 0.03	1.23,^75^ 1.72,^79^ 1.55^76^
Propane	298 K and 1.13 bar	0.31 ± 0.02	0.441,^93^ 0.945,^73^ 0.81,^94^ 1.13^95^
Propylene		0.384 ± 0.002	0.627,^93^ 1.218,^73^ 1.10,^94^ 1.36^95^
Propylene/Propane		1.24 ± 0.08	1.26,^93^ 1.29,^73^ 1.36,^94^ 1.21^95^

a At 308 K and 2 bar.

**Figure 10:**
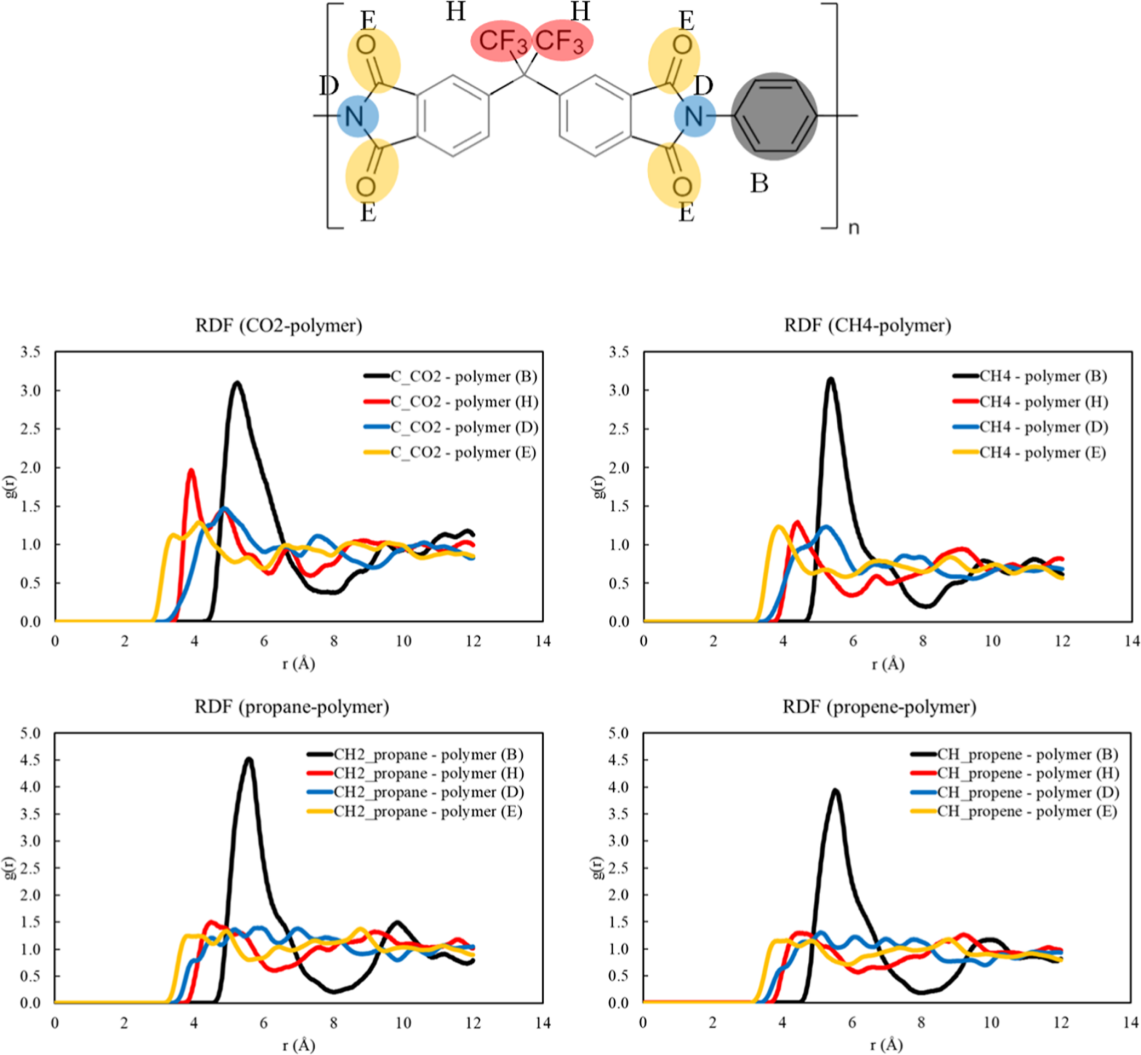
RDF of polymer (B, H, D, and E bead types shown in the chemical structure) and adsorbates central atom of CO_2_, CH_4_, propane, and propylene simulated using the TraPPE-UA force field.

The RDF is a powerful tool for analyzing molecular simulation results, providing detailed insight into the spatial distribution of atoms and molecules around a reference particle (here, polymer beads). In the context of sorption in polymeric membranes, the RDF can reveal preferential locations of sorbate molecules relative to specific groups in the polymer. The RDF figure set in Figure 10 visually represents these preferred sorption locations. Each peak in an RDF plot corresponds to a preferred distance between the sorbate and the bead. The peak height indicates the relative probability of finding a sorbate at that distance. Therefore, the nearest peaks in the RDF plots represent the most probable locations for sorbate molecules in the polymer matrix. This information is helpful in analyzing and modeling sorption and diffusivity computations. For all of the adsorbates, bead type E (−OH group) is in proximity to the adsorbates. The highest peak is observed with super bead type B (diamine monomer). Interactions between these beads and the adsorbate are one of the keys, besides the free volume of the polymer, to determining sorption capabilities. The preferential sorption trend agrees with other atomistic-based simulation work.^96^

A preliminary investigation was performed regarding the diffusion of propylene and propane in 6FDA-DAM, which has been shown to have selectivity toward propylene. Here, the structure obtained after GCMC/*NpT* adsorption cycles of propylene in 6FDA-DAM was used to estimate the diffusion property, reflecting the experimental uptake at 300 K and 1 bar. For propane, the same equilibrated structure was used, with the number of molecules inserted adjusted according to the calculated solubility selectivity to ensure a representative system loading. Simulations in the *NpT* ensemble were run for 1 μ s at three different temperatures (300, 500, and 700 K). The results show good agreement with experimental literature values, with a calculated propylene diffusion coefficient at 300 K of (0.97 ± 0.04) × 10^–9^ cm^2^/s and propane diffusion coefficient of (0.160 ± 0.005) × 10^–9^ cm^2^/s, which is of the same order of magnitude as the reported experimental measurements.^73^ The predicted diffusion selectivity is 6.1 ± 0.3, which is in reasonable agreement with the experimental values of 11.4^75^ and 8.8.^97^ At higher temperatures, the polymer expands and the pore size distribution shifts toward higher values, allowing propane and propylene to diffuse faster with a corresponding reduction in selectivity. The calculated diffusion selectivities of propylene/propane at 500 and 700 K are 1.3 ± 0.7 and 1.4 ± 0.5, respectively. This reduction in selectivity occurs because the polymer matrix becomes less effective at separating molecules by size at higher temperatures. As the temperature increases, the polymer chains gain greater mobility, and the material expands thermally, which weakens its ability to distinguish between molecules based on their size differences.

An additional property studied in this work was the bulk modulus, which accounts for the material’s response to hydrostatic pressure. It serves as an indicator of polymers’ compressibility and inherent stiffness. Bulk modulus can be estimated by monitoring the volume change as a function of hydrostatic pressure according to the formula
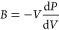
1

**Figure 11:**
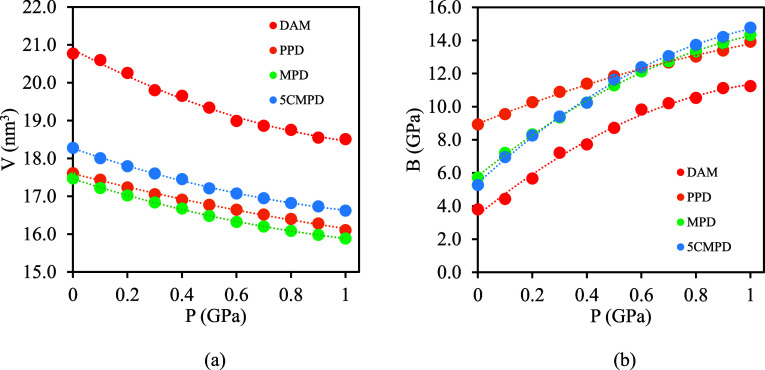
(a) Change of volume as a function of the hydrostatic pressure of 6FDA-DAM, PPD, MPD, and 5CMPD PIs, and (b) the pressure dependence of bulk modulus (B). All data correspond to molecular simulations performed here.

**Figure 12:**
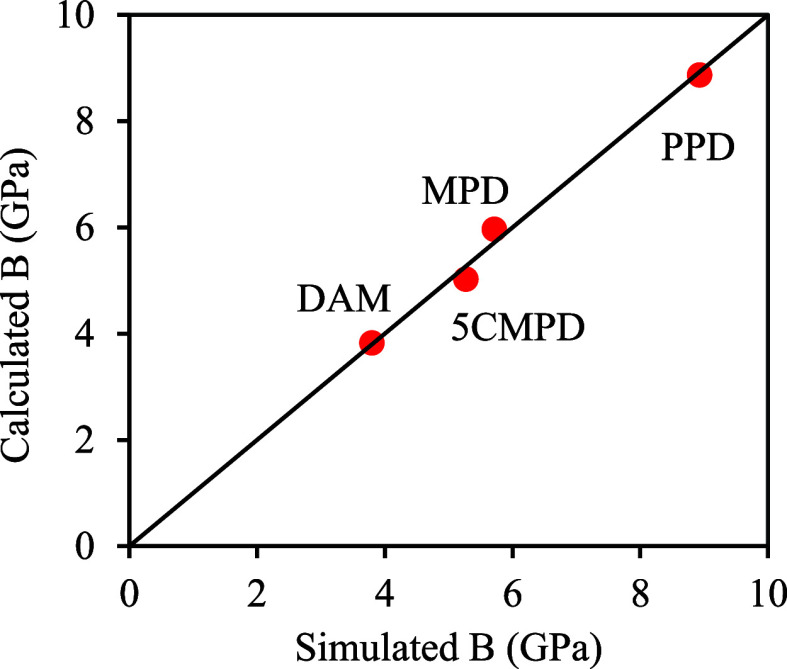
Comparison of model predictions using eq 2 and simulation results for the bulk modulus of selected polymers 6FDA-[PPD, MPD, DAM, 5CMPD].

A series of molecular simulations in the *NpT* ensemble were carried out to estimate the volume change as a function of pressure to measure the stiffness of the 6FDA-DAM, PPD, MPD, and 5CMPD polymers. Figure 11 shows the volume change as the pressure increases at 300 K. The results show that the bulk moduli, at 1 bar, calculated using eq 1, for DAM, PPD, MPD, and 5CMPD are equal to 3.8, 8.9, 5.7, and 5.3 GPa, which lie in the range of PIs bulk moduli. To model the bulk modulus, an expression that includes CED as an indicative of the material’s strength of intermolecular forces was used, inspired by a previous literature work.^98^ According to literature data, the bulk modulus accounts for the contribution of intermolecular forces.^98^ Also, an angle term was included to adjust the intermolecular flexibility. The expression provided by eq 2
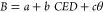
2resulted in an excellent agreement with the simulated bulk modulus at 1 bar as presented in Figure 12. Model coefficients are listed in Table S27. Both coefficients *B* and *C* possess positive values, suggesting that an increase in the catenation angle and a higher CED contribute to enhanced resistance to compressive stress.

## 4 Conclusions

In this work, we have successfully developed a novel set of CG force field parameters to study a family of PIs derived from 6FDA imide. These parameters have been effectively constructed by using direct atomistic descriptors. The models have demonstrated remarkable accuracy in predicting the specific volume of polymers studied. Interestingly, our study has revealed that parameters, specifically those associated with specific diamines, can be used to model various properties, particularly the density, using a multiple linear regression model. This finding highlights the potential of employing machine learning techniques to develop CG force fields and to predict the properties of various PI polymers.

Furthermore, we have determined key properties such as the radius of gyration, end-to-end distance, glass transition temperature (*T*_g_), pore size distribution, solubility, and diffusion coefficient of gases. The gas separation simulations in 6FDA-DAM showed excellent agreement with available experimental data on the solubility, diffusion, and overall selectivity for CO_2_, N_2_, O_2_, CH_4_, propylene, and propane. This accuracy suggests effective estimation of intermolecular interactions and free volume within the polymer matrix. Our investigation also explored the halogenation effects on diamines and introduced methods to estimate intermolecular interaction parameters. Furthermore, we examined how diamine variations influence the mechanical properties of the PI, focusing on the bulk modulus. The simulation results, supported by statistical modeling, strongly indicate that factors such as the catenation angle and CED are significant in determining the polymer’s compressibility. The findings of this research contribute to the understanding of PI behavior and open new possibilities for designing polymers with tuned properties for specific gas separation applications.

## Supporting Information Available

Detailed information and parameters of the force fields, validation of the TraPPE force field for small penetrants, additional details about simulation protocols and simulation variables, including the estimation of LJ parameters for larger CG beads, the combined effect of annealing cycles and cooling rate on the glassy polymeric structure, details on the configurational biased Monte Carlo simulations and results discussing the role of thermal moves dedicated to the polymer chains, calculations for the structural and diffusion properties of the PIs, detailed description of the statistical modeling performed in this work, and regression models for the other properties (density, *T*_g_, and CED) (PDF)

## Dedication

This paper is dedicated to Professor Athanassios Z. Panagiotopoulos, who has been an outstanding mentor, collaborator, and friend to the corresponding author of this work for more than 30 years. Professor Panagiotopoulos is a global leader in the development of advanced molecular simulation methods for complex systems, including the revolutionary Gibbs Ensemble Monte Carlo simulation method for the efficient simulation of phase equilibria for pure components and mixtures and many more.

## References

[ref1] Favvas E. P.; Katsaros F. K.; Papageorgiou S. K.; Sapalidis A. A.; Mitropoulos A. Ch. A Review of the Latest Development of Polyimide Based Membranes for CO 2 Separations. React. Funct. Polym. 2017, 120, 104–130. 10.1016/j.reactfunctpolym.2017.09.002.

[ref2] Ma X.-H.; Yang S.-Y. Polyimide Gas Separation Membranes. In Advanced Polyimide Materials; Elsevier, 2018; pp 257–322. 10.1016/B978-0-12-812640-0.00006-8.

[ref3] Sanaeepur H.; Ebadi Amooghin A.; Bandehali S.; Moghadassi A.; Matsuura T.; Van der Bruggen B. Polyimides in Membrane Gas Separation: Monomer’s Molecular Design and Structural Engineering. Prog. Polym. Sci. 2019, 91, 80–125. 10.1016/j.progpolymsci.2019.02.001.

[ref4] Sridhar S.; Veerapur R. S.; Patil M. B.; Gudasi K. B.; Aminabhavi T. M. Matrimid Polyimide Membranes for the Separation of Carbon Dioxide from Methane. J. Appl. Polym. Sci. 2007, 106 (3), 1585–1594. 10.1002/app.26306.

[ref5] Bae T.-H.; Lee J. S.; Qiu W.; Koros W. J.; Jones C. W.; Nair S. A High-Performance Gas-Separation Membrane Containing Submicrometer-Sized Metal-Organic Framework Crystals. Angew. Chem., Int. Ed. 2010, 49 (51), 9863–9866. 10.1002/anie.201006141. 21082645

[ref6] Liu G.; Chernikova V.; Liu Y.; Zhang K.; Belmabkhout Y.; Shekhah O.; Zhang C.; Yi S.; Eddaoudi M.; Koros W. J. Mixed Matrix Formulations with MOF Molecular Sieving for Key Energy-Intensive Separations. Nat. Mater. 2018, 17 (3), 283–289. 10.1038/s41563-017-0013-1. 29434309

[ref7] Wang S.; Li X.; Wu H.; Tian Z.; Xin Q.; He G.; Peng D.; Chen S.; Yin Y.; Jiang Z.; Guiver M. D. Advances in High Permeability Polymer-Based Membrane Materials for CO_2_ Separations. Energy Environ. Sci. 2016, 9 (6), 1863–1890. 10.1039/C6EE00811A.

[ref8] Khdhayyer M.; Bushell A. F.; Budd P. M.; Attfield M. P.; Jiang D.; Burrows A. D.; Esposito E.; Bernardo P.; Monteleone M.; Fuoco A.; Clarizia G.; Bazzarelli F.; Gordano A.; Jansen J. C. Mixed Matrix Membranes Based on MIL-101 Metal–Organic Frameworks in Polymer of Intrinsic Microporosity PIM-1. Sep. Purif. Technol. 2019, 212, 545–554. 10.1016/j.seppur.2018.11.055.

[ref9] Pechar T.; Kim S.; Vaughan B.; Marand E.; Tsapatsis M.; Jeong H.; Cornelius C. Fabrication and Characterization of Polyimide–Zeolite L Mixed Matrix Membranes for Gas Separations. J. Membr. Sci. 2006, 277 (1–2), 195–202. 10.1016/j.memsci.2005.10.029.

[ref10] Park S.; Cho K. Y.; Jeong H.-K. Polyimide/ZIF-7 Mixed-Matrix Membranes: Understanding the *in Situ* Confined Formation of the ZIF-7 Phases inside a Polymer and Their Effects on Gas Separations. J. Mater. Chem. A 2020, 8 (22), 11210–11217. 10.1039/D0TA02761H.

[ref11] Jiang H.; Li T.; Bai L.; Han J.; Zhang X.; Dong H.; Zeng S.; Luo S.; Zhang X. Polyimide/Ionic Liquids Hybrid Membranes with NH_3_ -Philic Channels for Ammonia-Based CO_2_ Separation Processes. ACS Appl. Mater. Interfaces 2023, 15 (44), 51204–51214. 10.1021/acsami.3c12200. 37874939

[ref12] Mulder M. Basic Principles of Membrane Technology; Springer Netherlands: Dordrecht, 1996 10.1007/978-94-009-1766-8.

[ref13] Baker R. W. Membrane Technology and Applications; John Wiley & Sons, Ltd: Chichester, UK, 2004 10.1002/0470020393.

[ref14] Han Y.; Ho W. S. W. Polymeric Membranes for CO2 Separation and Capture. J. Membr. Sci. 2021, 628, 119244 10.1016/j.memsci.2021.119244.

[ref15] Harmandaris V. A.; Mavrantzas V. G.; Theodorou D. N. Atomistic Molecular Dynamics Simulation of Polydisperse Linear Polyethylene Melts. Macromolecules 1998, 31 (22), 7934–7943. 10.1021/ma980698p.

[ref16] Harmandaris V. A.; Adhikari N. P.; van der Vegt N. F. A.; Kremer K. Hierarchical Modeling of Polystyrene: From Atomistic to Coarse-Grained Simulations. Macromolecules 2006, 39 (19), 6708–6719. 10.1021/ma0606399.

[ref17] Gartner T. E.; Jayaraman A. Modeling and Simulations of Polymers: A Roadmap. Macromolecules 2019, 52 (3), 755–786. 10.1021/acs.macromol.8b01836.

[ref18] Barrat J.-L.; Baschnagel J.; Lyulin A. Molecular Dynamics Simulations of Glassy Polymers. Soft Matter 2010, 6 (15), 3430–3446. 10.1039/b927044b.

[ref19] Neyertz S.; Brown D.; Pandiyan S.; van der Vegt N. F. A. Carbon Dioxide Diffusion and Plasticization in Fluorinated Polyimides. Macromolecules 2010, 43 (18), 7813–7827. 10.1021/ma1010205.

[ref20] Hölck O.; Böhning M.; Heuchel M.; Siegert M. R.; Hofmann D. Gas Sorption Isotherms in Swelling Glassy Polymers—Detailed Atomistic Simulations. J. Membr. Sci. 2013, 428, 523–532. 10.1016/j.memsci.2012.10.023.

[ref21] Han J.; Gee R. H.; Boyd R. H. Glass Transition Temperatures of Polymers from Molecular Dynamics Simulations. Macromolecules 1994, 27 (26), 7781–7784. 10.1021/ma00104a036.

[ref22] Vergadou N.; Theodorou D. N. Molecular Modeling Investigations of Sorption and Diffusion of Small Molecules in Glassy Polymers. Membranes 2019, 9 (8), 98 10.3390/membranes9080098. 31398889 PMC6723301

[ref23] Ge T.; Wang J.; Robbins M. O. Effects of Coarse-Graining on Molecular Simulations of Mechanical Properties of Glassy Polymers. Macromolecules 2021, 54 (5), 2277–2287. 10.1021/acs.macromol.0c02467.

[ref24] Panizon E.; Bochicchio D.; Monticelli L.; Rossi G. MARTINI Coarse-Grained Models of Polyethylene and Polypropylene. J. Phys. Chem. B 2015, 119 (25), 8209–8216. 10.1021/acs.jpcb.5b03611. 26000469

[ref25] Rossi G.; Monticelli L.; Puisto S. R.; Vattulainen I.; Ala-Nissila T. Coarse-Graining Polymers with the MARTINI Force-Field: Polystyrene as a Benchmark Case. Soft Matter 2011, 7 (2), 698–708. 10.1039/C0SM00481B.

[ref26] Pandiyan S.; Parandekar P. V.; Prakash O.; Tsotsis T. K.; Basu S. Systematic Coarse Graining of a High-Performance Polyimide. Macromol. Theory Simul. 2015, 24 (5), 513–520. 10.1002/mats.201500009.

[ref27] Sudarkodi V.; Sooraj K.; Nair N. N.; Basu S.; Parandekar P. V.; Sinha N. K.; Prakash O.; Tsotsis T. Mechanical Response of Two Polyimides through Coarse-Grained Molecular Dynamics Simulations. Model Simul Mat Sci. Eng. 2018, 26 (2), 025013 10.1088/1361-651X/aa9ee4.

[ref28] Hu C.; Lu T.; Guo H. Developing a Transferable Coarse-Grained Model for the Prediction of Thermodynamic, Structural, and Mechanical Properties of Polyimides at Different Thermodynamic State Points. J. Chem. Inf. Model. 2019, 59 (5), 2009–2025. 10.1021/acs.jcim.8b00887. 30920827

[ref29] Wen C.; Odle R.; Cheng S. Coarse-Grained Molecular Dynamics Modeling of a Branched Polyetherimide. Macromolecules 2021, 54 (1), 143–160. 10.1021/acs.macromol.0c01440.

[ref30] Chakrabarty A.; Cagin T. Coarse Grain Modeling of Polyimide Copolymers. Polymer 2010, 51 (12), 2786–2794. 10.1016/j.polymer.2010.03.060.

[ref31] Volgin I. V.; Larin S. V.; Lyulin A. V.; Lyulin S. V. Coarse-Grained Molecular-Dynamics Simulations of Nanoparticle Diffusion in Polymer Nanocomposites. Polymer 2018, 145, 80–87. 10.1016/j.polymer.2018.04.058.

[ref32] Harmandaris V. A.; Adhikari N. P.; van der Vegt N. F. A.; Kremer K.; Mann B. A.; Voelkel R.; Weiss H.; Liew C. Ethylbenzene Diffusion in Polystyrene: United Atom Atomistic/Coarse Grained Simulations and Experiments. Macromolecules 2007, 40 (19), 7026–7035. 10.1021/ma070201o.

[ref33] Bates F. S.; Hillmyer M. A.; Lodge T. P.; Bates C. M.; Delaney K. T.; Fredrickson G. H. Multiblock Polymers: Panacea or Pandora’s Box?. Polymer 2012, 336 (6080), 434–440. 10.1126/science.1215368. 22539713

[ref34] Yang R. T. Gas Separation by Adsorption Processes; Butterworths: Boston, 1987 10.1016/B978-0-409-90004-0.50013-1.

[ref35] Martin M. G.; Siepmann J. I. Transferable Potentials for Phase Equilibria. 1. United-Atom Description of n-Alkanes. J. Phys. Chem. B 1998, 102 (14), 2569–2577. 10.1021/jp972543+.

[ref36] Siu S. W. I.; Pluhackova K.; Böckmann R. A. Optimization of the OPLS-AA Force Field for Long Hydrocarbons. J. Chem. Theory Comput. 2012, 8 (4), 1459–1470. 10.1021/ct200908r. 26596756

[ref37] Zhu M.; Deng T.; Dong L.; Chen J.; Dang Z. Review of Machine Learning-driven Design of Polymer-based Dielectrics. IET Nanodielectrics 2022, 5 (1), 24–38. 10.1049/nde2.12029.

[ref38] Ye H.; Xian W.; Li Y. Machine Learning of Coarse-Grained Models for Organic Molecules and Polymers: Progress, Opportunities, and Challenges. ACS Omega 2021, 6 (3), 1758–1772. 10.1021/acsomega.0c05321. 33521417 PMC7841771

[ref39] Ricci E.; Vergadou N. Integrating Machine Learning in the Coarse-Grained Molecular Simulation of Polymers. J. Phys. Chem. B 2023, 127 (11), 2302–2322. 10.1021/acs.jpcb.2c06354. 36888553

[ref40] Carbone P.; Varzaneh H. A. K.; Chen X.; Müller-Plathe F. Transferability of Coarse-Grained Force Fields: The Polymer Case. J. Chem. Phys. 2008, 128 (6), 064904 10.1063/1.2829409. 18282071

[ref41] Lin E.; You X.; Kriegel R. M.; Moffitt R. D.; Batra R. C. Atomistic to Coarse Grained Simulations of Diffusion of Small Molecules into Polymeric Matrix. Comput. Mater. Sci. 2017, 138, 448–461. 10.1016/j.commatsci.2017.07.011.

[ref42] Monticelli L.; Kandasamy S. K.; Periole X.; Larson R. G.; Tieleman D. P.; Marrink S.-J. The MARTINI Coarse-Grained Force Field: Extension to Proteins. J. Chem. Theory Comput. 2008, 4 (5), 819–834. 10.1021/ct700324x. 26621095

[ref43] Marrink S. J.; Risselada H. J.; Yefimov S.; Tieleman D. P.; de Vries A. H. The MARTINI Force Field: Coarse Grained Model for Biomolecular Simulations. J. Phys. Chem. B 2007, 111 (27), 7812–7824. 10.1021/jp071097f. 17569554

[ref44] Barbosa G. D.; Turner C. H. Martini Coarse-Grained Model for Poly(Alkylimidazolium) Ionenes and Applications in Aromatic Compound Extraction. Macromolecules 2022, 55 (1), 26–34. 10.1021/acs.macromol.1c01932.

[ref45] Abraham M. J.; Murtola T.; Schulz R.; Páll S.; Smith J. C.; Hess B.; Lindahl E. GROMACS: High Performance Molecular Simulations through Multi-Level Parallelism from Laptops to Supercomputers. SoftwareX 2015, 1–2, 19–25. 10.1016/j.softx.2015.06.001.

[ref46] de Jong D. H.; Singh G.; Bennett W. F. D.; Arnarez C.; Wassenaar T. A.; Schäfer L. V.; Periole X.; Tieleman D. P.; Marrink S. J. Improved Parameters for the Martini Coarse-Grained Protein Force Field. J. Chem. Theory Comput. 2013, 9 (1), 687–697. 10.1021/ct300646g. 26589065

[ref47] Marrink S. J.; de Vries A. H.; Tieleman D. P. Lipids on the Move: Simulations of Membrane Pores, Domains, Stalks and Curves. Biochim. Biophys. Acta, Biomembr. 2009, 1788 (1), 149–168. 10.1016/j.bbamem.2008.10.006. 19013128

[ref48] Papavasileiou K. D.; Peristeras L. D.; Bick A.; Economou I. G. Molecular Dynamics Simulation of Pure n -Alkanes and Their Mixtures at Elevated Temperatures Using Atomistic and Coarse-Grained Force Fields. J. Phys. Chem. B 2019, 123 (29), 6229–6243. 10.1021/acs.jpcb.9b02840. 31251061

[ref49] Papavasileiou K. D.; Peristeras L. D.; Bick A.; Economou I. G. Molecular Dynamics Simulation of the *n* -Octacosane–Water Mixture Confined in Graphene Mesopores: Comparison of Atomistic and Coarse-Grained Calculations and the Effect of Catalyst Nanoparticle. Energy Fuels 2021, 35 (5), 4313–4332. 10.1021/acs.energyfuels.0c04151.

[ref50] Papavasileiou K. D.; Peristeras L. D.; Boulougouris G. C.; Economou I. G. Coarse-Grained Molecular Dynamics Simulation of Cobalt Nanoparticle in the *n* -Octacosane–Water Mixture: The Effect of Water Concentration and Nanoparticle Size. J. Phys. Chem. C 2022, 126 (32), 13975–13985. 10.1021/acs.jpcc.2c03681.

[ref51] Gatsiou C. A.; Bick A.; Krokidis X.; Economou I. G. Atomistic and Coarse-Grained Simulations of Bulk Amorphous Amylose Above and Below the Glass Transition. Macromolecules 2022, 55 (8), 2999–3010. 10.1021/acs.macromol.1c01925.

[ref52] Marrink S. J.; Tieleman D. P. Perspective on the Martini Model. Chem. Soc. Rev. 2013, 42 (16), 6801–6822. 10.1039/c3cs60093a. 23708257

[ref53] Müller-Plathe F. Coarse-Graining in Polymer Simulation: From the Atomistic to the Mesoscopic Scale and Back. ChemPhysChem 2002, 3 (9), 754–769. 10.1002/1439-7641(20020916)3:9<754::AID-CPHC754>3.0.CO;2-U. 12436902

[ref54] Zhao M.; Sampath J.; Alamdari S.; Shen G.; Chen C.-L.; Mundy C. J.; Pfaendtner J.; Ferguson A. L. MARTINI-Compatible Coarse-Grained Model for the Mesoscale Simulation of Peptoids. J. Phys. Chem. B 2020, 124 (36), 7745–7764. 10.1021/acs.jpcb.0c04567. 32790381

[ref55] Souza P. C. T.; Alessandri R.; Barnoud J.; Thallmair S.; Faustino I.; Grünewald F.; Patmanidis I.; Abdizadeh H.; Bruininks B. M. H.; Wassenaar T. A.; Kroon P. C.; Melcr J.; Nieto V.; Corradi V.; Khan H. M.; Domański J.; Javanainen M.; Martinez-Seara H.; Reuter N.; Best R. B.; Vattulainen I.; Monticelli L.; Periole X.; Tieleman D. P.; de Vries A. H.; Marrink S. J. Martini 3: A General Purpose Force Field for Coarse-Grained Molecular Dynamics. Nat. Methods 2021, 18 (4), 382–388. 10.1038/s41592-021-01098-3. 33782607 PMC12554258

[ref56] Potter T. D.; Barrett E. L.; Miller M. A. Automated Coarse-Grained Mapping Algorithm for the Martini Force Field and Benchmarks for Membrane–Water Partitioning. J. Chem. Theory Comput. 2021, 17 (9), 5777–5791. 10.1021/acs.jctc.1c00322. 34472843 PMC8444346

[ref57] Pan T.; Dutta S.; Sing C. E. Interaction Potential for Coarse-Grained Models of Bottlebrush Polymers. J. Chem. Phys. 2022, 156 (1), 014903 10.1063/5.0076507. 34998351

[ref58] Martin M. G.; Siepmann J. I. Transferable Potentials for Phase Equilibria. 1. United-Atom Description of n -Alkanes. J. Phys. Chem. B 1998, 102 (97), 2569–2577. 10.1021/jp972543+.

[ref59] Vazquez-Salazar L. I.; Selle M.; de Vries A. H.; Marrink S. J.; Souza P. C. T. Martini Coarse-Grained Models of Imidazolium-Based Ionic Liquids: From Nanostructural Organization to Liquid–Liquid Extraction. Green Chem. 2020, 22 (21), 7376–7386. 10.1039/D0GC01823F.

[ref60] Sun H.; Mumby S. J.; Maple J. R.; Hagler A. T. An Ab Initio CFF93 All-Atom Force Field for Polycarbonates. J. Am. Chem. Soc. 1994, 116 (7), 2978–2987. 10.1021/ja00086a030.

[ref61] Pandiyan S.; Brown D.; van der Vegt N. F. A.; Neyertz S. Atomistic Models of Three Fluorinated Polyimides in the Amorphous State. J. Polym. Sci., Part B:Polym. Phys. 2009, 47 (12), 1166–1180. 10.1002/polb.21717.

[ref62] Ahmad M. Z.; Navarro M.; Lhotka M.; Zornoza B.; Téllez C.; de Vos W. M.; Benes N. E.; Konnertz N. M.; Visser T.; Semino R.; Maurin G.; Fila V.; Coronas J. Enhanced Gas Separation Performance of 6FDA-DAM Based Mixed Matrix Membranes by Incorporating MOF UiO-66 and Its Derivatives. J. Membr. Sci. 2018, 558, 64–77. 10.1016/j.memsci.2018.04.040.

[ref63] Liu Z.; Cao Y.; Qiu W.; Kim J.; Campbell Z. S.; Schlosser S.; Koros W. J. Insights on Influence of Polymer Molecular Weight on Gas Sorption, Transport and Plasticization in Glassy 6FDA-DAM Polyimide Membranes. J. Membr. Sci. 2024, 712, 123242 10.1016/j.memsci.2024.123242.

[ref64] Shah J. K.; Marin-Rimoldi E.; Mullen R. G.; Keene B. P.; Khan S.; Paluch A. S.; Rai N.; Romanielo L. L.; Rosch T. W.; Yoo B.; Maginn E. J. Cassandra: An Open Source Monte Carlo Package for Molecular Simulation. J. Comput. Chem. 2017, 38 (19), 1727–1739. 10.1002/jcc.24807. 28436594

[ref65] Zhang L.; Siepmann J. I. Direct Calculation of Henry’s Law Constants from Gibbs Ensemble Monte Carlo Simulations: Nitrogen, Oxygen, Carbon Dioxide and Methane in Ethanol. Theor. Chem. Acc. 2006, 115 (5), 391–397. 10.1007/s00214-005-0073-1.

[ref66] Wick C. D.; Martin M. G.; Siepmann J. I. Transferable Potentials for Phase Equilibria. 4. United-Atom Description of Linear and Branched Alkenes and Alkylbenzenes. J. Phys. Chem. B 2000, 104 (33), 8008–8016. 10.1021/jp001044x.

[ref67] Potoff J. J.; Siepmann J. I. Vapor–Liquid Equilibria of Mixtures Containing Alkanes, Carbon Dioxide, and Nitrogen. AIChE J. 2001, 47 (7), 1676–1682. 10.1002/aic.690470719.

[ref68] Jia Y.; Lu Y.; Yang H.; Chen Y.; Hillman F.; Wang K.; Liang C. Z.; Zhang S. Control of Microporous Structure in Conjugated Microporous Polymer Membranes for Post-Combustion Carbon Capture. Adv. Funct. Mater. 2024, 34 (45), 2407499 10.1002/adfm.202407499.

[ref69] Phan B. K.; Shen K.-H.; Gurnani R.; Tran H.; Lively R.; Ramprasad R. Gas Permeability, Diffusivity, and Solubility in Polymers: Simulation-Experiment Data Fusion and Multi-Task Machine Learning. npj Comput. Mater. 2024, 10 (1), 186 10.1038/s41524-024-01373-9.

[ref70] Rzepiela A. J.; Louhivuori M.; Peter C.; Marrink S. J. Hybrid Simulations: Combining Atomistic and Coarse-Grained Force Fields Using Virtual Sites. Phys. Chem. Chem. Phys. 2011, 13 (22), 10437 10.1039/c0cp02981e. 21494747

[ref71] Wassenaar T. A.; Ingólfsson H. I.; Prieß M.; Marrink S. J.; Schäfer L. V. Mixing MARTINI: Electrostatic Coupling in Hybrid Atomistic–Coarse-Grained Biomolecular Simulations. J. Phys. Chem. B 2013, 117 (13), 3516–3530. 10.1021/jp311533p. 23406326

[ref72] Zervopoulou E.; Mavrantzas V. G.; Theodorou D. N. A New Monte Carlo Simulation Approach for the Prediction of Sorption Equilibria of Oligomers in Polymer Melts: Solubility of Long Alkanes in Linear Polyethylene. J. Chem. Phys. 2001, 115 (6), 2860–2875. 10.1063/1.1383050.

[ref73] Shimazu A.; Miyazaki T.; Maeda M.; Ikeda K. Relationships between the Chemical Structures and the Solubility, Diffusivity, and Permselectivity of Propylene and Propane in 6FDA-Based Polyimides. J. Polym. Sci., Part B:Polym. Phys. 2000, 38 (19), 2525–2536. 10.1002/1099-0488(20001001)38:19<2525::AID-POLB40>3.0.CO;2-2.

[ref74] Afzal M. A. F.; Cheng C.; Hachmann J. Combining First-Principles and Data Modeling for the Accurate Prediction of the Refractive Index of Organic Polymers. J. Chem. Phys. 2018, 148 (24), 241712 10.1063/1.5007873. 29960320

[ref75] Tanaka K.; Okano M.; Toshino H.; Kita H.; Okamoto K.-I. Effect of Methyl Substituents on Permeability and Permselectivity of Gases in Polyimides Prepared from Methyl-Substituted Phenylenediamines. J. Polym. Sci., Part B:Polym. Phys. 1992, 30 (8), 907–914. 10.1002/polb.1992.090300813.

[ref76] Fritsch D.; Peinemann K.-V. Novel Highly Permselective 6F-Poly(Amide-Imide)s as Membrane Host for Nano-Sized Catalysts. J. Membr. Sci. 1995, 99 (1), 29–38. 10.1016/0376-7388(94)00201-9.

[ref77] Matsui S.; Sato H.; Nakagawa T. Effects of Low Molecular Weight Photosensitizer and UV Irradiation on Gas Permeability and Selectivity of Polyimide Membrane. J. Membr. Sci. 1998, 141 (1), 31–43. 10.1016/S0376-7388(97)00286-X.

[ref78] Zornoza B.; Téllez C.; Coronas J.; Esekhile O.; Koros W. J. Mixed Matrix Membranes Based on 6FDA Polyimide with Silica and Zeolite Microsphere Dispersed Phases. AIChE J. 2015, 61 (12), 4481–4490. 10.1002/aic.15011.

[ref79] Yeom C. K.; Lee J. M.; Hong Y. T.; Choi K. Y.; Kim S. C. Analysis of Permeation Transients of Pure Gases through Dense Polymeric Membranes Measured by a New Permeation Apparatus. J. Membr. Sci. 2000, 166 (1), 71–83. 10.1016/S0376-7388(99)00252-5.

[ref80] Qiu W.; Xu L.; Chen C.-C.; Paul D. R.; Koros W. J. Gas Separation Performance of 6FDA-Based Polyimides with Different Chemical Structures. Polymer 2013, 54 (22), 6226–6235. 10.1016/j.polymer.2013.09.007.

[ref81] Esekhile O.; Qiu W.; Koros W. J. Permeation of Butane Isomers through 6FDA-DAM Dense Films. J. Polym. Sci., Part B:Polym. Phys. 2011, 49 (22), 1605–1620. 10.1002/polb.22351.

[ref82] Gasteiger J.; Marsili M. A New Model for Calculating Atomic Charges in Molecules. Tetrahedron Lett. 1978, 19 (34), 3181–3184. 10.1016/S0040-4039(01)94977-9.

[ref83] Falkovich S. G.; Lyulin S. V.; Nazarychev V. M.; Larin S. V.; Gurtovenko A. A.; Lukasheva N. V.; Lyulin A. V. Influence of the Electrostatic Interactions on Thermophysical Properties of Polyimides: Molecular-Dynamics Simulations. J. Polym. Sci., Part B:Polym. Phys. 2014, 52 (9), 640–646. 10.1002/polb.23460.

[ref84] White R. P.; Lipson J. E. G. Polymer Free Volume and Its Connection to the Glass Transition. Macromolecules 2016, 49 (11), 3987–4007. 10.1021/acs.macromol.6b00215.

[ref85] Ghosh A.; Mistri E. A.; Banerjee S. Fluorinated Polyimides. In Handbook of Specialty Fluorinated Polymers; Elsevier, 2015; pp 97–185 10.1016/B978-0-323-35792-0.00003-9.

[ref86] Abdulhamid M. A.; Ma X.; Ghanem B. S.; Pinnau I. Synthesis and Characterization of Organo-Soluble Polyimides Derived from Alicyclic Dianhydrides and a Dihydroxyl-Functionalized Spirobisindane Diamine. ACS Appl. Polym. Mater. 2019, 1 (1), 63–69. 10.1021/acsapm.8b00036.

[ref87] Willems T. F.; Rycroft C. H.; Kazi M.; Meza J. C.; Haranczyk M. Algorithms and Tools for High-Throughput Geometry-Based Analysis of Crystalline Porous Materials. Microporous Mesoporous Mater. 2012, 149 (1), 134–141. 10.1016/j.micromeso.2011.08.020.

[ref88] Flory P. J. Statistical Mechanics of Chain Molecules; Wiley: New York, 1969.

[ref89] Neyertz S.; Brown D. An Optimized Fully-Atomistic Procedure to Generate Glassy Polymer Films for Molecular Dynamics Simulations. Comput. Mater. Sci. 2020, 174, 109499 10.1016/j.commatsci.2019.109499.

[ref90] Tanis I.; Brown D.; Neyertz S. J.; Heck R.; Mercier R. A Comparison of Homopolymer and Block Copolymer Structure in 6FDA-Based Polyimides. Phys. Chem. Chem. Phys. 2014, 16 (42), 23044–23055. 10.1039/C4CP03039G. 25247609

[ref91] Burns R. L.; Koros W. J. Structure–Property Relationships for Poly(Pyrrolone-Imide) Gas Separation Membranes. Macromolecules 2003, 36 (7), 2374–2381. 10.1021/ma0259261.

[ref92] Liu Y.; Liu Z.; Liu G.; Qiu W.; Bhuwania N.; Chinn D.; Koros W. J. Surprising Plasticization Benefits in Natural Gas Upgrading Using Polyimide Membranes. J. Membr. Sci. 2020, 593, 117430 10.1016/j.memsci.2019.117430.

[ref93] Burns R. L.; Koros W. J. Defining the Challenges for C3H6/C3H8 Separation Using Polymeric Membranes. J. Membr. Sci. 2003, 211 (2), 299–309. 10.1016/S0376-7388(02)00430-1.

[ref94] Knebel A.; Bavykina A.; Datta S. J.; Sundermann L.; Garzon-Tovar L.; Lebedev Y.; Durini S.; Ahmad R.; Kozlov S. M.; Shterk G.; Karunakaran M.; Carja I. D.; Simic D.; Weilert I.; Klüppel M.; Giese U.; Cavallo L.; Rueping M.; Eddaoudi M.; Caro J.; Gascon J. Solution Processable Metal–Organic Frameworks for Mixed Matrix Membranes Using Porous Liquids. Nat. Mater. 2020, 19 (12), 1346–1353. 10.1038/s41563-020-0764-y. 32778813

[ref95] Liu Y.; Chen Z.; Liu G.; Belmabkhout Y.; Adil K.; Eddaoudi M.; Koros W. Conformation-Controlled Molecular Sieving Effects for Membrane-Based Propylene/Propane Separation. Adv. Mater. 2019, 31 (14), 1807513 10.1002/adma.201807513. 30768815

[ref96] Velioğlu S.; Ahunbay M. G.; Tantekin-Ersolmaz S. B. Investigation of CO2-Induced Plasticization in Fluorinated Polyimide Membranes via Molecular Simulation. J. Membr. Sci. 2012, 417–418, 217–227. 10.1016/j.memsci.2012.06.043.

[ref97] Tanaka K.; Taguchi A.; Hao J.; Kita H.; Okamoto K. Permeation and Separation Properties of Polyimide Membranes to Olefins and Paraffins. J. Membr. Sci. 1996, 121 (2), 197–207. 10.1016/S0376-7388(96)00182-2.

[ref98] Tabor D. The Bulk Modulus of Rubber. Polymer 1994, 35 (13), 2759–2763. 10.1016/0032-3861(94)90304-2.

