# Supplementary material for: Polyimide Polymer Simulations through Coarse-Grained Modeling: Prediction of Structure, Physical Properties, and Gas Separation Properties
Source: J Phys Chem B. 2025 Apr 30;129(19):4765–80. doi: 10.1021/acs.jpcb.4c04595 (PMC12086849; doi:10.1021/acs.jpcb.4c04595)
Supplement: Supplementary file 1 — jp4c04595_si_001.pdf [file jp4c04595_si_001.pdf]

# Supporting Information

# Polyimide polymer simulations through coarse-grained modeling: Prediction of structure, physical properties and gas separation properties

Amro M. O. Mohamed,[1,(a)] Ioannis G. Economou,[1,*] and Hae-Kwon Jeong[2,3]

[1]*Chemical Engineering Program, Texas A&M University at Qatar, PO Box 23874, Doha, Qatar*

[2]*Artie McFerrin Department of Chemical Engineering, Texas A&M University, 3122 TAMU, College Station, TX 77843-3122, United States*

[3]*Department of Materials Science and Engineering, Texas A&M University, 3122 TAMU, College Station, TX 77843-3122, United States*

*Corresponding author at ioannis.economou@qatar.tamu.edu

[(a)]*Current address: Qatar Environment and Energy Research Institute, Hamad Bin Khalifa University, Doha, Qatar*

The supporting information document contains detailed information and parameters of the coarse grained force fields for each of the polymers examined in this work. It also reports PCFF parameters used to model halogenated diamines. Details about simulation protocols and simulation variables are provided.

**Section 1: Force field parameters to model polyimides using CG simulations and force field parameters for penetrates using TraPPE force field**

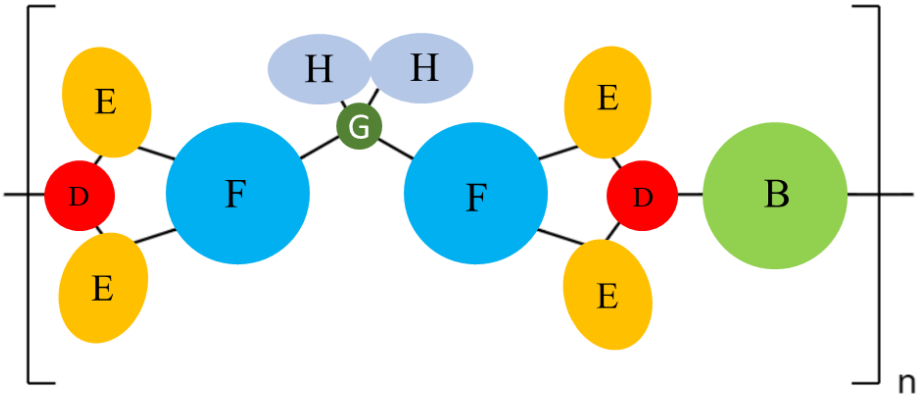


Figure S1. Bead identification for the polyimide. Bead B represents the diamine co-monomer.

Table S1: Masses of beads used in the simulations.

<table>
<tr><th colspan="2">Bead</th><th>Mass (g mol<sup>-1</sup>)</th><th colspan="2">Bead</th><th>Mass (g mol<sup>-1</sup>)</th></tr>
<tr><td colspan="2">D</td><td>15</td><td rowspan="8">B</td><td>25DPX</td><td>104.1</td></tr>
<tr><td colspan="2">E</td><td>28</td><td>DAM</td><td>118</td></tr>
<tr><td colspan="2">F</td><td>75</td><td>TMPPD</td><td>132.2</td></tr>
<tr><td colspan="2">G</td><td>12</td><td>5CMPD</td><td>110.5</td></tr>
<tr><td colspan="2">H</td><td>69</td><td>DBA</td><td>120.0</td></tr>
<tr><td rowspan="3">B</td><td>PPD</td><td>75.7</td><td>24DPD</td><td>92.0</td></tr>
<tr><td>MPD</td><td>75.7</td><td>24DAD</td><td>106.1</td></tr>
<tr><td>25DAT</td><td>90.1</td><td>15ND</td><td>126.0</td></tr>
</table>

<u>Force field functional forms:</u>

1- Bond stretching:

$$V_b(r_{ij}) = \frac{1}{2}k_{ij}^{b}(r_{ij} - b_{ij})^2 \quad \text{(S1)}$$

2- Bond angle bending:

$$V_a(\theta_{ijk}) = \frac{1}{2}k_{ijk}^{\theta}(\theta_{ij} - \theta_{ij}^{0})^2 \quad \text{(S2)}$$

3- Dihedral angle distortion:

$$V_{rb}(\phi_{ijkl}) = \sum_{n=0}^{5} C_n(\cos(\psi))^n \ ; \psi = \phi - 180 \quad \text{(S3)}$$

4- Non-bonded interactions:

$$U_{LJ}(r_1 \ldots r_n) = \sum_{i,j} 4\varepsilon_{ij}\left[\left(\frac{\sigma_{ij}}{r_{ij}}\right)^{12} - \left(\frac{\sigma_{ij}}{r_{ij}}\right)^{6}\right] \quad \text{(S4)}$$

$$\sigma_{ij} = \left(\frac{\sigma_{ii} + \sigma_{jj}}{2}\right) - s_{ij} \ ; s_{ij} = 0.01 \quad \text{(S5)}$$

$s_{ij}$ is the softness parameter. The parameter assumes that bigger beads should be softer than smaller ones. $\varepsilon_{ij}$ parameters based on the fully revised interaction matrix by Martini 3 model and scaled for beads with more than 4 atoms (larger than regular bead defined in Martini 3). Details on the scaling procedure relative to the size of the larger beads is available in the following section.

The nonbonded parameters are found using the following procedure.

For a given bead, the size of the bead is assigned by an estimation of the surface area ($A$) of the atomistic structure using defined van der Waals radii of the atoms of the bead. The following equation is used to calculate $\sigma_i$.

$$\sigma_i = \frac{1}{\sqrt[6]{2}} \times \sqrt{\frac{A}{\pi}} \quad \text{(S6)}$$

To determine the energy parameters $\varepsilon_i$ and $\varepsilon_{ij}$, the following steps are used. The selection of bead type is done according to the Martini 3 framework (Table S4). Here, we are considering the diamine and the entire benzene molecule, each as a single bead. This results in a large bead, larger than the Regular bead in the Martini 3 framework. As such we had to develop a procedure to extend the energy parameters tailored to capture the size effect beyond the size of regular bead. This is accomplished by statistically modeling the interaction table parameters (Supplementary Tables S4-S12: Martini 3 interaction matrix in Martini 3 SI document[1]), relative to the size of the beads. First, the self-interactions of tiny (T), small (S) and regular (R) beads at each different interaction level are fitted using linear regression (the regression is used with the number of atoms in a given bead). Second, a similar linear fitting analysis is done for the cross-interaction. In order to extrapolate to larger bead sizes, the size of the new larger bead, $\sigma_i$, is converted to the number of atoms in a bead using another linear regression of size vs number of atoms of T, S and R beads of Martini 3. An example of this extension is BC5 used to model benzene ring in 6FDA imide (bead F in Figure S1). The resulting $\sigma_i$ and $\varepsilon_i$ from the procedure above are 5.40 Å and 4.368 kJ mol$^{-1}$, which compares well with single bead LJ benzene potential, determined from viscosity data, of 5.349 Å and 3.428 kJ mol$^{-1}$.[2]

## 1.1. Force field parameters for penetrants using TraPPE force field

Table S2: Non-bonded interaction parameters for the small penetrates in this study.

| Molecule | Pseudo-atom | Type | $\varepsilon/k_B$ (K) | $\sigma$(Å) | q (e) |
|---|---|---|---|---|---|
| Carbon dioxide (TraPPE-small) | C | O=[C]=O | 27 | 2.80 | 0.70 |
| | O | [O]=C=O | 79 | 3.05 | -0.35 |
| | O | O=C=[O] | 79 | 3.05 | -0.35 |
| Methane (TraPPE-UA) | CH4 | CH4 | 148 | 3.73 | 0.00 |
| Propane (UA) | CH3 | [CH3]-CHx | 98 | 3.75 | 0.00 |
| | CH2 | CHx-[CH2]-CHx | 47 | 3.95 | 0.00 |
| | CH3 | [CH3]-CHx | 98 | 3.75 | 0.00 |
| Propene (TraPPE-UA) | CH3 | [CH3]-CHx | 98 | 3.75 | 0.00 |
| | CH | CHx=[CH](sp2)-CHy(sp3) | 47 | 3.73 | 0.00 |
| | CH2 | [CH2]=CHx | 85 | 3.675 | 0.00 |
| Nitrogen (TraPPE-small) | N | [N]-N | 36 | 3.310 | -0.482 |
| | M | N-[M]-N | 0 | 0.00 | 0.964 |
| | N | [N]-N | 36 | 3.310 | -0.482 |
| Oxygen (TraPPE-small) | O | [O]=O | 49 | 3.02 | -0.113 |
| | M | O=[M]=O | 0 | 0.00 | 0.226 |
| | O | [O]=O | 49 | 3.02 | -0.113 |

Table S3: Bonded interaction parameters for the small penetrants in this study.

| Bond Lengths | | |
|---|---|---|
| Molecule | Type | Length (Å) |
| Carbon dioxide (TraPPE-small) | O=(C=O) | 1.16 |
| Propane (UA) | CH3-CH2 | 1.54 |
| Propene (TraPPE-UA) | CH3-CH | 1.54 |
| | CH-CH2 | 1.33 |
| Nitrogen (TraPPE-small) | N-M | 0.55 |
| Oxygen (TraPPE-small) | O-M | 0.605 |
| Bond Angles | | |
| Molecule | Type | $\theta$(°) |
| Carbon dioxide (TraPPE-small) | O=(C)=O | 180 |
| Propane (UA) | CHx-(CH2)-CHy | 114 |
| Propene (TraPPE-UA) | CHx=(CH)-CHx | 119.70 |
| | CH-CH2 | 1.33 |
| Nitrogen (TraPPE-small) | N-(M)-N | 180 |
| Oxygen (TraPPE-small) | O=(M)=O | 180 |

### 1.2. Validation of the TraPPE force field in modeling small penetrants

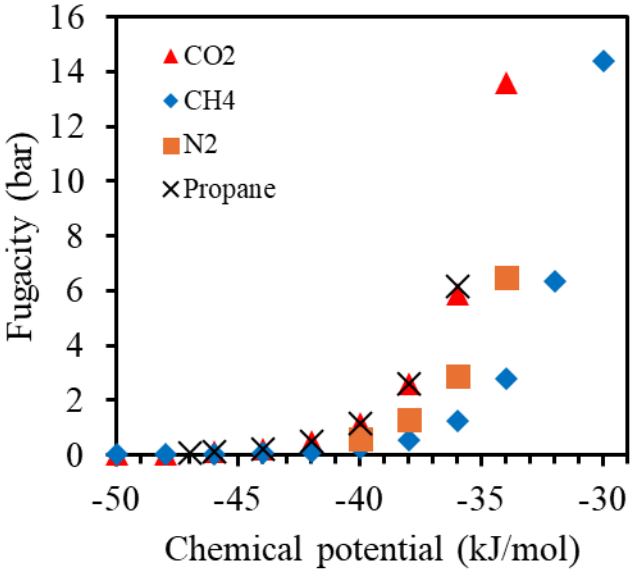


Figure S2: Fugacity versus shifted chemical potential for $CO_2$, $CH_4$, $N_2$ and propane.

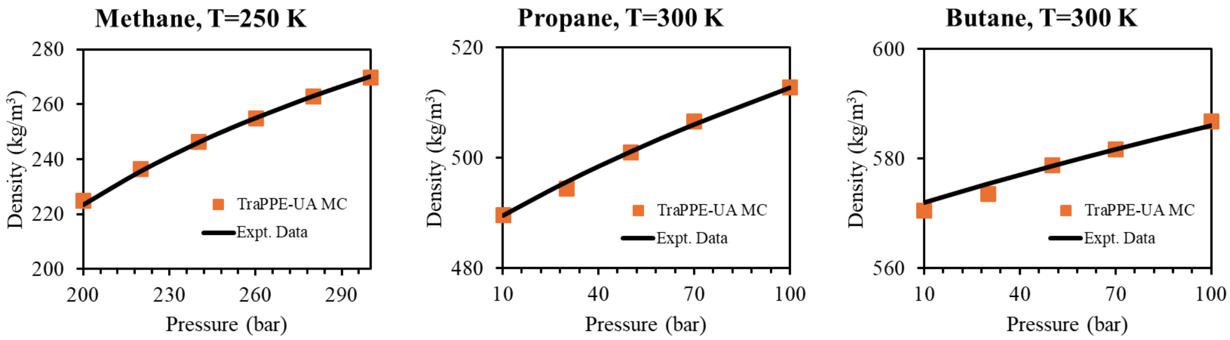


Figure S3: Density isotherms (methane, propane and *n*-butane). Experimental data and NPT MC simulations (points) using the TraPPE force field.

Table S4: Types of Martini beads used in the simulations.

| Type | | Martini-3 Bead type |
|---|---|---|
| D | | N6d |
| E | | N6a |
| H | | X4e |
| F | | C5 |
| G | | C1 |
| B | PPD | C5 |
| | MPD | C5 |
| | 25DAT | C5 |
| | 25DPX | C4-C5 |
| | TrMPD | C4-C5 |
| | TMPPD | C4 |
| | 5CMPD | C5-X3 |
| | DBA | Q5N |
| | 24DPD | T6 |

Table S5: Bonded interactions for the CG polyimides (see eq S1).

| Type | | $k_{ij}^{b}$ (*kJ mol⁻¹ nm⁻²*) | $b_{ij}$ (Å) |
|---|---|---|---|
| D – E | | 325694 | 1.85 |
| E – F | | 345124 | 3.32 |
| F – G | | 744542 | 2.92 |
| G – H | | 403687 | 2.02 |
| B – D | PPD | 744542 | 2.800 |
| | MPD | 744542 | 2.815 |
| | 25DAT | 744542 | 2.810 |
| | 25DPX | 744542 | 2.810 |
| | DAM | 744542 | 2.836 |
| | TMPPD | 744542 | 2.740 |
| | 5CMPD | 744542 | 3.036 |
| | DBA | 744542 | 3.500 |
| | 24DPD | 744542 | 2.980 |
| | 24DAD | 744542 | 3.356 |
| | 15ND | 744542 | 2.800 |

Table S6: Bonded interactions for the CG polyimides (see eq S2).

| Type | | $k_{ijk}^{\theta}$ (*kJ mol$^{-1}$ rad$^{-2}$*) | $\theta_{ij}^{0}$ |
|---|---|---|---|
| D – E – F | | 2954 | 74 |
| E – F – G | | 750 | 116 |
| F – G – H | | 2547.36 | 121 |
| F – G – F | | 1770.64 | 90 |
| E – F – E | | 5089 | 63 |
| E – D – E | | 1263.3 | 146 |
| E – D – B (TMPPD) | | 633.13 | 106 (90) |
| D – B – D | PPD | 1195.87 | 179.97 |
| | MPD | 1195.87 | 120 |
| | 25DAT | 1195.87 | 161.0 |
| | 25DPX | 1195.87 | 180 |
| | DAM | 1195.87 | 119.47 |
| | TMPPD | 1195.87 | 177.00 |
| | 5CMPD | 1195.87 | 110.0 |
| | DBA | 1195.87 | 84.21 |
| | 24DPD | 1195.87 | 104 |
| | 24DAD | 1195.87 | 92.5 |
| | 15ND | 1195.87 | 180 |

Table S7: Bonded interactions for the CG polyimides (see eq. S3).

| Type | $C_1$ (*kJ mol$^{-1}$*) | $C_2$ (*kJ mol$^{-1}$*) | $C_3$ (*kJ mol$^{-1}$*) | $C_4$ (*kJ mol$^{-1}$*) | $C_5$ (*kJ mol$^{-1}$*) | $C_6$ (*kJ mol$^{-1}$*) |
|---|---|---|---|---|---|---|
| F – E – D – B | 4.01 | -6.98 | -3.33 | 9.58 | 2.14 | -5.44 |
| E – D – B – D | 0.92 | -0.13 | -2.92 | 0.17 | 2.40 | 0.02 |
| G – F – E – D | 0.86 | -0.16 | 4.33 | 2.20 | -3.82 | -3.06 |
| F – G – F – E | 0.41 | 1.26 | 2.88 | -5.04 | -2.41 | 4.67 |
| H – G – F – E | 0.58 | -1.15 | -0.25 | 2.20 | 0.18 | -1.09 |

Table S8: Non-bonded interactions for the CG polyimides (PPD and MPD).

| Type 1 | Type 2 | $\varepsilon_{ij}$ (*kJ mol*$^{-1}$) | $\sigma_{ij}$ (*Å*) |
|---|---|---|---|
| B | D | 2.415 | 4.260 |
| B | E | 2.415 | 4.260 |
| B | F | 4.681 | 5.260 |
| B | G | 1.642 | 4.090 |
| B | H | 3.450 | 4.610 |
| B | B | 4.552 | 5.320 |
| D | E | 3.018 | 3.400 |
| D | F | 2.365 | 4.300 |
| D | G | 0.310 | 3.355 |
| D | H | 2.102 | 3.650 |
| D | D | 2.015 | 3.400 |
| E | F | 2.365 | 4.300 |
| E | G | 0.310 | 3.355 |
| E | H | 1.755 | 3.650 |
| E | E | 2.015 | 3.400 |
| F | G | 1.610 | 4.130 |
| F | H | 3.377 | 4.650 |
| F | F | 4.368 | 5.400 |
| G | H | 1.224 | 3.480 |
| G | G | 1.066 | 3.060 |
| H | H | 2.223 | 4.100 |

Table S9: Non-bonded interactions for the CG polyimides (25DAT).

| Type 1 | Type 2 | $\varepsilon_{ij}$ (*kJ mol*$^{-1}$) | $\sigma_{ij}$ (*Å*) |
|---|---|---|---|
| B | D | 2.397 | 4.500 |
| B | E | 2.397 | 4.500 |
| B | F | 4.854 | 5.500 |
| B | G | 1.698 | 4.330 |
| B | H | 3.579 | 4.850 |
| B | B | 4.928 | 5.800 |
| D | E | 3.018 | 3.400 |
| D | F | 2.365 | 4.300 |
| D | G | 0.310 | 3.355 |
| D | H | 2.102 | 3.650 |
| D | D | 2.015 | 3.400 |
| E | F | 2.365 | 4.300 |
| E | G | 0.310 | 3.355 |
| E | H | 1.755 | 3.650 |
| E | E | 2.015 | 3.400 |
| F | G | 1.610 | 4.130 |
| F | H | 3.377 | 4.650 |
| F | F | 4.368 | 5.400 |
| G | H | 1.224 | 3.480 |
| G | G | 1.066 | 3.060 |
| H | H | 2.223 | 4.100 |

Table S10: Non-bonded interactions for the CG polyimides (25DPX).

| Type 1 | Type 2 | $\varepsilon_{ij}$ (*kJ mol*$^{-1}$) | $\sigma_{ij}$ (*Å*) |
|---|---|---|---|
| B | D | 2.457 | 4.769 |
| B | E | 2.457 | 4.768 |
| B | F | 5.179 | 5.768 |
| B | G | 1.803 | 4.598 |
| B | H | 3.821 | 5.118 |
| B | B | 5.588 | 6.336 |
| D | E | 3.018 | 3.400 |
| D | F | 2.365 | 4.300 |
| D | G | 0.310 | 3.355 |
| D | H | 2.102 | 3.650 |
| D | D | 2.015 | 3.400 |
| E | F | 2.365 | 4.300 |
| E | G | 0.310 | 3.355 |
| E | H | 1.755 | 3.650 |
| E | E | 2.015 | 3.400 |
| F | G | 1.610 | 4.130 |
| F | H | 3.377 | 4.650 |
| F | F | 4.368 | 5.400 |
| G | H | 1.224 | 3.480 |
| G | G | 1.066 | 3.060 |
| H | H | 2.223 | 4.100 |

Table S11: Non-bonded interactions for the CG polyimides (DAM).

| Type 1 | Type 2 | $\varepsilon_{ij}$ (*kJ mol*$^{-1}$) | $\sigma_{ij}$ (*Å*) |
|---|---|---|---|
| B | D | 2.790 | 5.048 |
| B | E | 2.790 | 5.048 |
| B | F | 6.131 | 5.988 |
| B | G | 2.109 | 4.818 |
| B | H | 4.529 | 5.338 |
| B | B | 7.717 | 6.775 |
| D | E | 3.018 | 3.400 |
| D | F | 2.365 | 4.300 |
| D | G | 0.310 | 3.355 |
| D | H | 2.102 | 3.650 |
| D | D | 2.015 | 3.400 |
| E | F | 2.365 | 4.300 |
| E | G | 0.310 | 3.355 |
| E | H | 1.755 | 3.650 |
| E | E | 2.015 | 3.400 |
| F | G | 1.610 | 4.130 |
| F | H | 3.377 | 4.650 |
| F | F | 4.368 | 5.400 |
| G | H | 1.224 | 3.480 |
| G | G | 1.066 | 3.060 |
| H | H | 2.223 | 4.100 |

Table S12: Non-bonded interactions for the CG polyimides (TMPPD).

| Type 1 | Type 2 | $\varepsilon_{ij}$ (*kJ mol*$^{-1}$) | $\sigma_{ij}$ (*Å*) |
|---|---|---|---|
| B | D | 2.947 | 5.150 |
| B | E | 2.947 | 5.150 |
| B | F | 6.492 | 6.151 |
| B | G | 1.992 | 4.980 |
| B | H | 4.798 | 5.500 |
| B | B | 8.634 | 7.100 |
| D | E | 3.018 | 3.400 |
| D | F | 2.365 | 4.300 |
| D | G | 0.310 | 3.355 |
| D | H | 2.102 | 3.650 |
| D | D | 2.015 | 3.400 |
| E | F | 2.365 | 4.300 |
| E | G | 0.310 | 3.355 |
| E | H | 1.755 | 3.650 |
| E | E | 2.015 | 3.400 |
| F | G | 1.610 | 4.130 |
| F | H | 3.377 | 4.650 |
| F | F | 4.368 | 5.400 |
| G | H | 1.224 | 3.480 |
| G | G | 1.066 | 3.060 |
| H | H | 2.223 | 4.100 |

Table S13: Non-bonded interactions for the CG polyimides (5CMPD).

| Type 1 | Type 2 | $\varepsilon_{ij}$ (*kJ mol*$^{-1}$) | $\sigma_{ij}$ (*Å*) |
|---|---|---|---|
| B | D | 2.475 | 4.445 |
| B | E | 2.475 | 4.445 |
| B | F | 4.755 | 5.445 |
| B | G | 1.681 | 4.275 |
| B | H | 3.583 | 4.795 |
| B | B | 4.781 | 5.689 |
| D | E | 3.018 | 3.400 |
| D | F | 2.365 | 4.300 |
| D | G | 0.310 | 3.355 |
| D | H | 2.102 | 3.650 |
| D | D | 2.015 | 3.400 |
| E | F | 2.365 | 4.300 |
| E | G | 0.310 | 3.355 |
| E | H | 1.755 | 3.650 |
| E | E | 2.015 | 3.400 |
| F | G | 1.610 | 4.130 |
| F | H | 3.377 | 4.650 |
| F | F | 4.368 | 5.400 |
| G | H | 1.224 | 3.480 |
| G | G | 1.066 | 3.060 |
| H | H | 2.223 | 4.100 |

Table S14: Non-bonded interactions for the CG polyimides (DBA).

| Type 1 | Type 2 | $\varepsilon_{ij}$ (*kJ mol*$^{-1}$) | $\sigma_{ij}$ (*Å*) |
|---|---|---|---|
| B | D | 3.501 | 4.600 |
| B | E | 3.973 | 4.600 |
| B | F | 3.060 | 4.600 |
| B | G | 1.730 | 4.400 |
| B | H | 2.140 | 4.950 |
| B | B | 6.184 | 6.000 |
| D | E | 3.018 | 3.400 |
| D | F | 2.365 | 4.300 |
| D | G | 0.310 | 3.355 |
| D | H | 2.102 | 3.650 |
| D | D | 2.015 | 3.400 |
| E | F | 2.365 | 4.300 |
| E | G | 0.310 | 3.355 |
| E | H | 1.755 | 3.650 |
| E | E | 2.015 | 3.400 |
| F | G | 1.610 | 4.130 |
| F | H | 3.377 | 4.650 |
| F | F | 4.368 | 5.400 |
| G | H | 1.224 | 3.480 |
| G | G | 1.066 | 3.060 |
| H | H | 2.223 | 4.100 |

Table S15: Non-bonded interactions for the CG polyimides (24DPD).

| Type 1 | Type 2 | $\varepsilon_{ij}$ (*kJ mol*$^{-1}$) | $\sigma_{ij}$ (*Å*) |
|---|---|---|---|
| B | D | 2.550 | 4.295 |
| B | E | 3.150 | 4.295 |
| B | F | 4.619 | 5.295 |
| B | G | 1.650 | 4.215 |
| B | H | 3.970 | 4.645 |
| B | B | 4.890 | 5.390 |
| D | E | 3.018 | 3.400 |
| D | F | 2.365 | 4.300 |
| D | G | 0.310 | 3.355 |
| D | H | 2.102 | 3.650 |
| D | D | 2.015 | 3.400 |
| E | F | 2.365 | 4.300 |
| E | G | 0.310 | 3.355 |
| E | H | 1.755 | 3.650 |
| E | E | 2.015 | 3.400 |
| F | G | 1.610 | 4.130 |
| F | H | 3.377 | 4.650 |
| F | F | 4.368 | 5.400 |
| G | H | 1.224 | 3.480 |
| G | G | 1.066 | 3.060 |
| H | H | 2.223 | 4.100 |

Table S16: Non-bonded interactions for the CG polyimides (24DAD).

| Type 1 | Type 2 | $\varepsilon_{ij}$ (*kJ mol*$^{-1}$) | $\sigma_{ij}$ (*Å*) |
|---|---|---|---|
| B | D | 2.637 | 4.514 |
| B | E | 2.637 | 4.514 |
| B | F | 4.844 | 5.514 |
| B | G | 1.416 | 4.344 |
| B | H | 3.295 | 4.864 |
| B | B | 5.296 | 5.827 |
| D | E | 3.018 | 3.400 |
| D | F | 2.365 | 4.300 |
| D | G | 0.310 | 3.355 |
| D | H | 2.102 | 3.650 |
| D | D | 2.015 | 3.400 |
| E | F | 2.365 | 4.300 |
| E | G | 0.310 | 3.355 |
| E | H | 1.755 | 3.650 |
| E | E | 2.015 | 3.400 |
| F | G | 1.610 | 4.130 |
| F | H | 3.377 | 4.650 |
| F | F | 4.368 | 5.400 |
| G | H | 1.224 | 3.480 |
| G | G | 1.066 | 3.060 |
| H | H | 2.223 | 4.100 |

Table S17: Non-bonded interactions for the CG polyimides (15ND).

| Type 1 | Type 2 | $\varepsilon_{ij}$ (*kJ mol*$^{-1}$) | $\sigma_{ij}$ (*Å*) |
|---|---|---|---|
| B | D | 3.040 | 4.820 |
| B | E | 3.040 | 4.820 |
| B | F | 5.773 | 5.820 |
| B | G | 1.848 | 4.650 |
| B | H | 3.654 | 5.170 |
| B | B | 5.692 | 6.444 |
| D | E | 3.018 | 3.400 |
| D | F | 2.365 | 4.300 |
| D | G | 0.310 | 3.355 |
| D | H | 2.102 | 3.650 |
| D | D | 2.015 | 3.400 |
| E | F | 2.365 | 4.300 |
| E | G | 0.310 | 3.355 |
| E | H | 1.755 | 3.650 |
| E | E | 2.015 | 3.400 |
| F | G | 1.610 | 4.130 |
| F | H | 3.377 | 4.650 |
| F | F | 4.368 | 5.400 |
| G | H | 1.224 | 3.480 |
| G | G | 1.066 | 3.060 |
| H | H | 2.223 | 4.100 |

## Section 2: Detailed results of the CG models

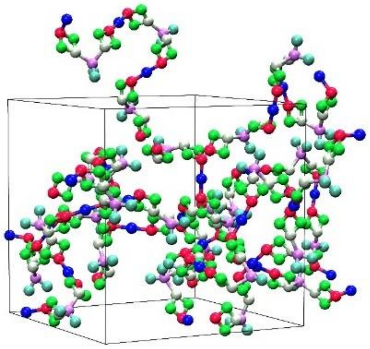

Figure S4. The initial configuration of the 6FDA-DAM glassy polymer using the new CG model (starting point is a 3 nm side cubic simulation box).

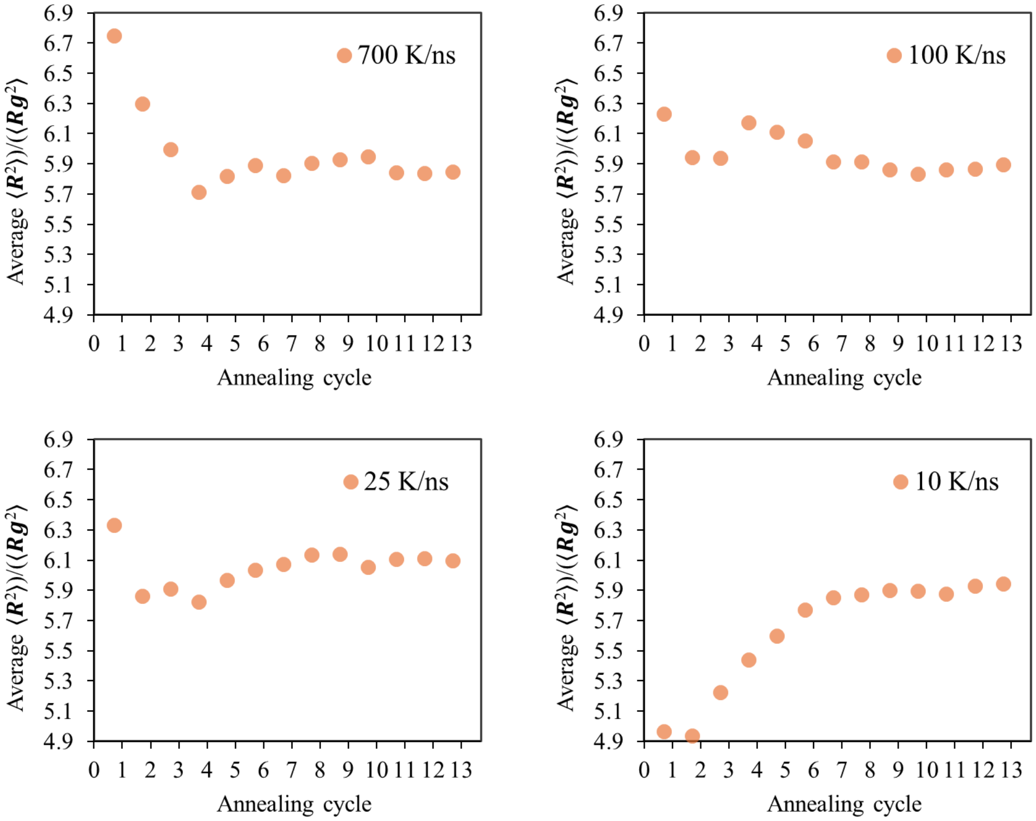


Figure S5. Running average of the ratio of the squared of end-to-end distance to the squared radius of gyration of polymer 6FDA-DAM using CG model in this work using up to 13 annealing cycles (using different cooling rates 700 K/ns to 10 K/ns).

**Section 3: Force field parameters to model polyimides using PCFF atomistic simulations and statistical parameters for prediction and fitting of various properties**

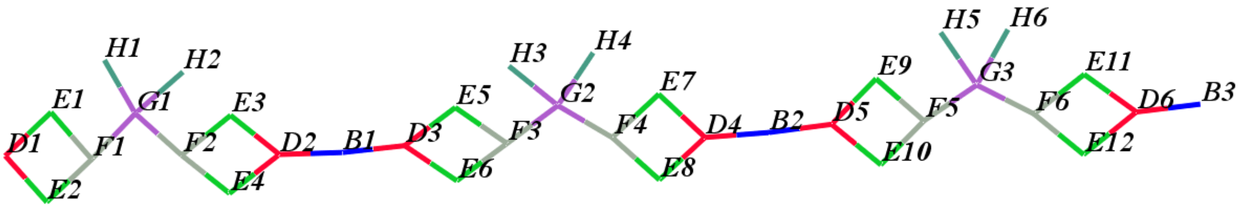


Figure S6. Atom types used to model 6FDA-based polyimides using PCFF force field.

Table S18: Charges used for the CG polyimides (trimer of 6FDA-DAM).

| Type | Charge (e) | Type | Charge (e) | Type | Charge (e) |
|---|---|---|---|---|---|
| D1 | -0.05007 | D3 | -0.14362 | D5 | -0.14362 |
| E1 | -0.03463 | E5 | -0.02687 | E9 | -0.02687 |
| E2 | -0.03464 | E6 | -0.02688 | E10 | -0.02688 |
| F1 | 0.10996 | F3 | 0.11117 | F5 | 0.11117 |
| G1 | 0.20952 | G2 | 0.20952 | G2 | 0.20952 |
| H1 | -0.09538 | H3 | -0.09538 | H5 | -0.09538 |
| H2 | -0.09538 | H4 | -0.09538 | H6 | -0.09538 |
| F2 | 0.11117 | F4 | 0.11117 | F6 | 0.11117 |
| E3 | -0.02687 | E7 | -0.02687 | E11 | -0.02687 |
| E4 | -0.02688 | E8 | -0.02688 | E12 | -0.02688 |
| D2 | -0.14362 | D4 | -0.14362 | D6 | -0.14367 |
| B1 | 0.15366 | B2 | 0.15366 | B3 | 0.07689 |

### 3.1. Variables used in the modeling work

Table S19: Values of variables used in the predictive modeling. Parameters $\varepsilon_1$ and $\varepsilon_2$ are the energy of interactions between diamine-$CF_3$ and diamine-N(imide).

| Polymer | Density ($kg\ m^{-3}$) | $T_g$ ($K$) | CED ($MJ\ m^{-3}$) | $\sigma$ ($nm$) | $l$ ($Å$) | $\varepsilon_1$ ($kJ\ mol^{-1}$) | $\varepsilon_2$ ($kJ\ mol^{-1}$) |
|---|---|---|---|---|---|---|---|
| PPD | 1.469 | 584 | 556 | 0.532001 | 2.8936 | 3.503 | 2.451 |
| MPD | 1.478 | 548 | 562 | 0.532001 | 2.9089 | 3.503 | 2.451 |
| 25DAT | 1.449 | 591 | 544 | 0.585358 | 3.1395 | 3.667 | 2.459 |
| DPX | 1.386 | 604 | 524 | 0.633622 | 2.9085 | 3.830 | 2.468 |
| Trmpd | 1.34 | 649 | 499 | 0.678288 | 2.9325 | 3.994 | 2.476 |
| TMPPD | 1.325 | 679 | 487 | 0.714311 | 2.9058 | 4.108 | 2.544 |
| 15ND | 1.425 | 551 | 546 | 0.636746 | 3.2105 | 3.885 | 2.711 |
| 5CMPD | 1.5 | 550 | 554 | 0.568894 | 3.5090 | 3.688 | 2.540 |
| DBA | 1.511 | 546 | 595 | 0.606913 | 3.7197 | 3.847 | 3.855 |
| 24DPD | 1.504 | 562 | 574 | 0.558009 | 3.4013 | 3.646 | 2.703 |
| 24DAD | 1.459 | 555 | 551 | 0.607223 | 3.7872 | 3.305 | 2.647 |

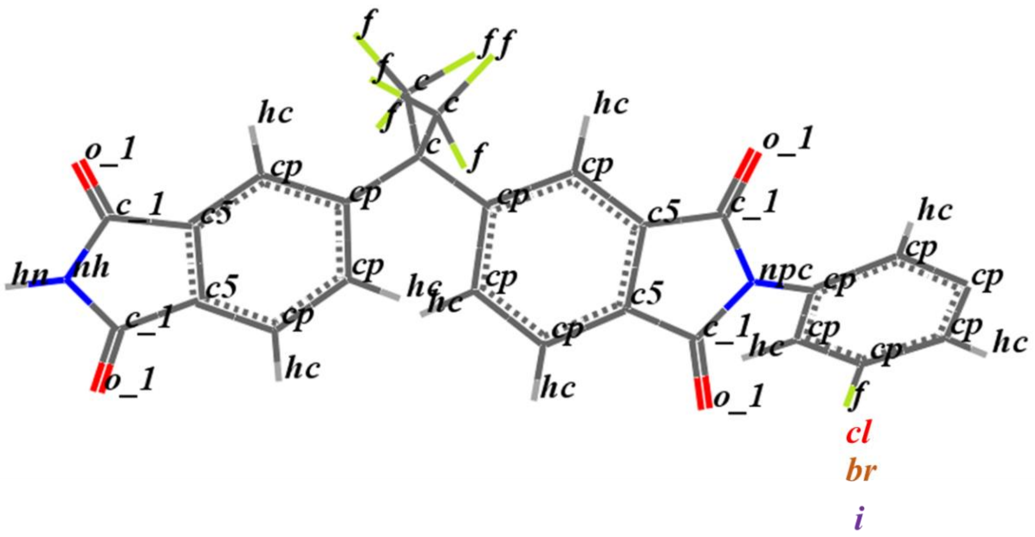


Figure S7. Atom types used to model 6FDA-based polyimides using PCFF force field

Table S20: Non-bonded interactions for the PCFF force field to model halogenated diamines.

| Type | $\varepsilon$ ($kJ\ mol^{-1}$) | $\sigma$ (Å) |
|---|---|---|
| hn | 0.0546 | 1.098 |
| f | 0.2512 | 3.2 |
| cp | 0.2688 | 4.01 |
| cp | 0.2268 | 4.01 |
| c5 | 0.2688 | 4.01 |
| c_1 | 0.5040 | 3.81 |
| hc | 0.0840 | 2.995 |
| o_1 | 1.1214 | 3.3 |
| nh | 0.5628 | 4.07 |
| npc | 0.5628 | 4.07 |
| cl | 1.6800 | 4.8 |
| br | 1.4654 | 4.3 |
| i | 0.9437 | 3.92 |

**Section 4: Statistical modeling of polymer properties: Density, $T_g$, CED and mechanical properties (bulk modulus)**

Several attempts were also run for polynomials and cross terms. The multiple linear models relate some variables to the property and identify outliers. We identified that the bead size used to model the co-monomer is a critical parameter in estimating the density and accessible volume. Other important parameters include the bond length connecting the co-monomers and the energy of interactions between selected beads on the 6FDA monomer (carbonyl group and start point of the monomer (N) sites). The following is the multiple linear regression model used to model the properties of the group of PIs:

$$Property\ (\rho, T_g, CED) = A + B\ \sigma + C\ l + D\ \varepsilon_1 + E\ \varepsilon_2 + F\ \theta + G\ MW_{monomer} \qquad \text{(S7)}$$

where, $\sigma$, $l$, $\varepsilon_1$, $\varepsilon_2$, and $\theta$ are the size of the diamine, the distance between imide and diamine beads, the energy of interactions between diamine-$CF_3$, diamine-N(imide), the catenation angle, and molecular weight of a single monomer, respectively. A, B, C, D, E, F and G are fitting parameters. Parameter values for the polymers are listed in Table S21.

The p-value is a crucial parameter in statistical modeling, assessing the degree of evidence against a null hypothesis. Each term's p-value tests the null hypothesis that the coefficient is equal to zero (no effect). A low p-value suggests that the null hypothesis can be rejected. The alpha level, a pre-defined threshold of significance, determines when the p-value is considered significant. This threshold, which is typically set as low as 0.05, delineates the boundary for statistical significance, with results below this level indicating that the observed data would be implausible under the null hypothesis. This study uses a backward elimination strategy with a 0.1 alpha level, corresponding to a confidence level is 90%, to ensure a more inclusive model during the variable selection

process. In addition, k-fold cross-validation is used to test the model's performance, reducing the risk of overfitting and providing a broader view of its predictive capabilities.

First, we utilize backward elimination based on the P-value of the variables in which the alpha level, the significance level, to remove is 0.10. According to this analysis, we eliminate $MW_{monomer}$ and $\theta$ terms as the P-values are 0.559 and 0.343, respectively. Afterwards, we use stepwise based on validation. Models are selected based on the forward selection with validation. The validation method used is the K-fold cross-validation method, where the data are divided into K random folds; here, K is assigned equal to the number of data points. In other words, the data is fitted on the expected data and then analyzed to determine how well the data fit for the hold-out fold. This process is repeated and rotated for every fold. The model is selected to find the model with the best 11-fold R-squared, which is calculated using the sums of squares errors for held-out data at every run and create an R-squared measure. It is important to note that the model selected should be of the least number of variables to highlight the importance of parameters. The model can be used to identify those parameters to model the CG parameters for new beads.

Figure S7(a) presents a compelling comparison between the experimental and predicted densities for several polymers, demonstrating the efficacy of our model, which is grounded in multiple linear regression. The model exhibits impressive agreement with the experimental data, with the most significant percentage error being a mere 0.85% (See residual plot in Figure S7(b)). This high accuracy level underscores our model’s robustness and potential utility in polymer research. The model was initially constructed using six variables. The maximum 11-fold stepwise R-squared is obtained without $\theta$ and $MW_{monomer}$.

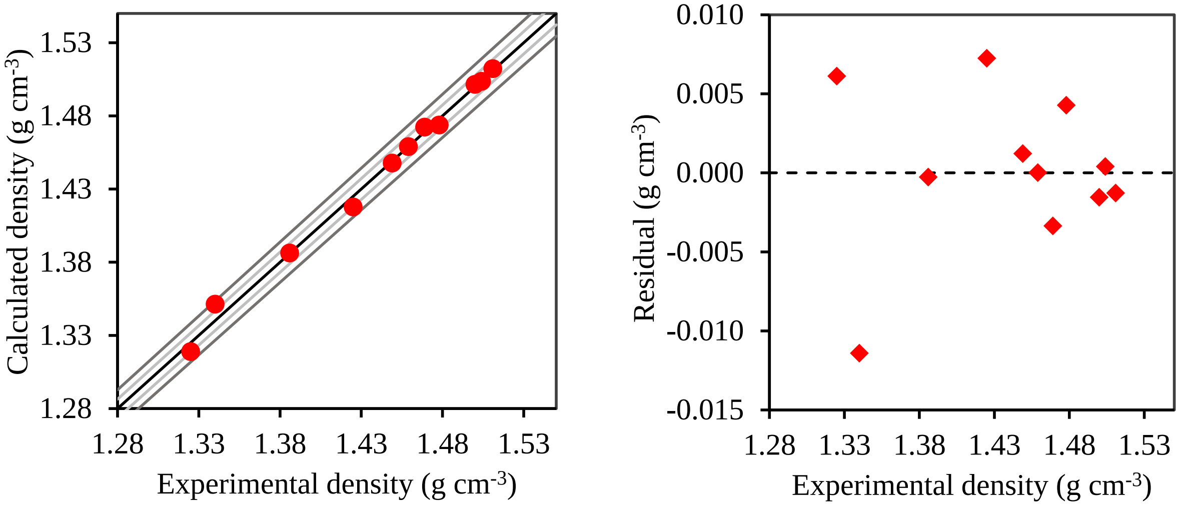


(a) (b)

Figure S8. (a) Comparison between experimental density and multiple regression model density. The polymers used are the ones listed in Table 1 in the main text. The continuous light and dark grey lines show 0.5% and 1% relative deviation. (b) The residual value of the calculated density with respect to experimental density.

The model parameters provide further evidence of its reliability from a statistical perspective. The multiple R-value of 0.9939 indicates a strong correlation between the predicted and experimental densities. The adjusted R-square value of 0.9898 considers the number of predictors in the model and confirms the significance of the five selected variables. Most importantly, the result predicted 11-fold stepwise (R-squared predicted = 91.83%) shows a remarkable statistical measure of the model's validity.

Table S21. Coefficient of the multiple linear regression model used to model density (model 1 and model 2). Model 2 ensures that a molecular weight term is included in the model.

| Coefficient | Variable | Model 1 | Model 2 |
|---|---|---|---|
| | | Value and standard error coefficient | |
| **A** | Constant | 1.50±0.01 | 1.88±0.01 |
| **B** | $\sigma$ | -1.09±0.06 | -1.30±0.04 |
| **C** | $l$ | 0.09±0.01 | 0.06±0.01 |
| **D** | $\epsilon_1$ | 0.02±0.01 | 0 |
| **E** | $\epsilon_2$ | 0.07±0.02 | 0 |
| **F** | $\theta$ | 0 | 0 |
| **G** | $MW_{monomer}$ | 0 | 1.43±0.02 |

It is essential to analyze these coefficients in Table S21 to understand and infer from the physics of these variables. The coefficients of the size and catenation angle are negative, indicating that we observe a decrease in mass density with the increase in the size of the bead and catenation angle. Also, the higher the distance and interaction energies with the diamine bead, the higher the density. The diamine molecular size emerged as the most significant predictor among the four variables, as indicated by its extremely low P-value. This statistical analysis, coupled with the fact that these variables are also used in simulation, strongly indicates that our model can be leveraged to design new polymers for specific applications.

Equations S8, S9 and S10 below provide the terms used to model CED and $T_g$, which provided the most accurate 11-fold R-squared. Equations S8 and S9 offer accurate predictions of the CED, a factor that influences the performance of polymeric membranes in gas separation applications. The first model, Equation S8, utilizes only two terms - the density of the polymer and $\varepsilon_2$ (the interaction energy between diamine-N(imide)) - yields an 11-fold prediction accuracy of 93.36%.

Notably, the density term carries the most statistical weight in this equation. The predictive accuracy can be further enhanced to 95.27% by incorporating the $C\rho^2$ term, as shown in Equation S9. Predicting the $T_g$ appears to be a more complex task, requiring the testing of several models to achieve a high prediction rate. The model outlined in Equation S10 demonstrates the best performance, with an 11-fold prediction accuracy of 89.49% and an R-squared value of 95.89%. The terms $\sigma_A$ and $\sigma_V$ in this equation correspond to the diamine molecular size, calculated from either the molecular surface area or the molecular volume, respectively. The inclusion of the catenation angle term highlights its significance in determining the conformational flexibility of the polymer chain. The model's predictive capacity is exemplified by its ability to discern the difference between PPD and MPD, which possess identical size parameters (σ) but distinct catenation angles (180° for PPD and 120° for MPD). The predicted $T_g$ values for PPD and MPD are 580 K and 568 K, whereas experimental values for PPD and MPD are 584 K and 548 K, respectively[3]. Coefficients for the models in equations S8 – S10 are listed in Tables S22 – S24.

$$CED^1 = A + B\rho + C\varepsilon_2 \tag{S8}$$

$$CED^2 = A + B\rho + C\rho^2 + D\varepsilon_2 \tag{S9}$$

$$T_g = A + B\sigma_A^3 + C\sigma_V^2 + D\theta \tag{S10}$$

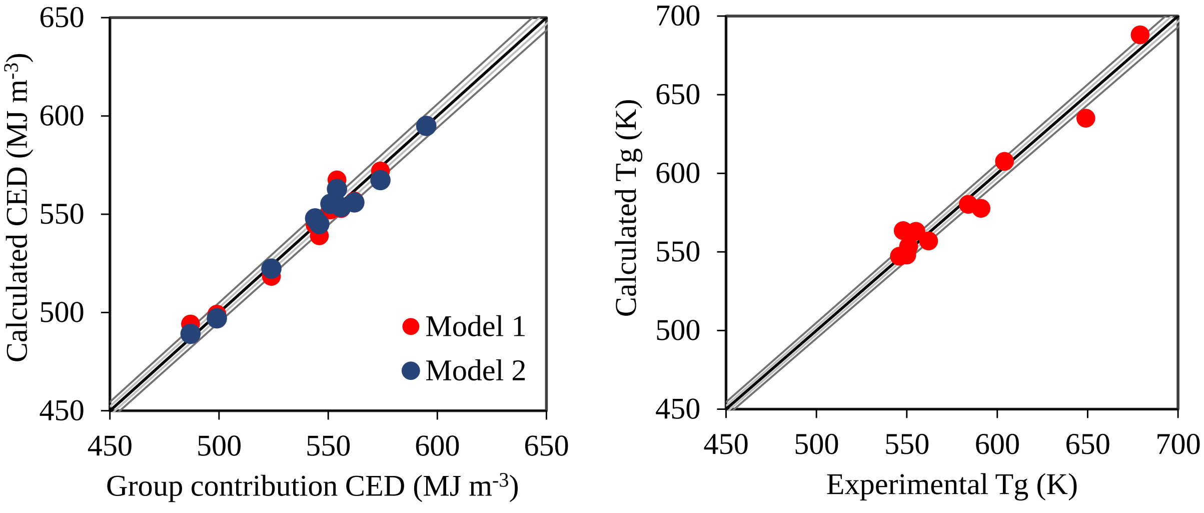


(a)

(b)

Figure S9. Comparison between (a) group contribution CED (as reported by Shimazu et al.[3]) and (b) experimental $T_g$[3] with using multiple linear regression models.

There are several notes to consider when dealing with the discussed models. Firstly, the models are based on linear relationships and do not account for potential interactions between the selected variables. Many properties of polymers, including free volume, $T_g$, and CED, are influenced by complex, non-linear relationships between various molecular features. The linear nature of our model may oversimplify these relationships, leading to less accurate predictions for these properties. Secondly, the model may not include all the necessary variables to predict these properties accurately. Polymers are complex materials, and many factors can influence their properties. While size, distance, and energy parameters were significant in predicting density, other properties may be more influenced by variables not included in our model, such as the charge of the bead and structural properties. Despite these limitations, our model provides a valuable starting point for developing more sophisticated models that can accurately predict a broader range

of polymer properties. Future work could incorporate non-linear relationships and additional variables into the model to improve its predictive power for free volume, $T_g$, and CED properties.

Table S22: model parameters for CED model 1 used in equation S8.

| Parameter | Value |
|---|---|
| A | -106.55 |
| B | 419.6 |
| C | 73.8 |

Table S23: model parameters for CED model 2 used in equation S9.

| Parameter | Value |
|---|---|
| A | -2764 |
| B | 4168 |
| C | -1326 |
| D | 96.5 |

Table S24: model parameters for $T_g$ model used in equation S10.

| Parameter | value |
|---|---|
| A | 1161 |
| B | 3263 |
| C | -4787 |
| D | 0.2789 |

**Section 5: Calculations for the structural and diffusion properties of PIs**

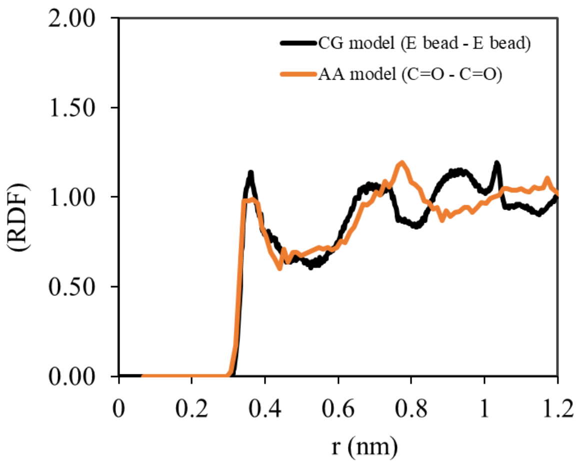


Figure S10. Inter-molecular radial distribution function (RDF) from atomistic (PCFF) and CG models for 6FDA-DAM. AA calculations refer to the RDF between C=O groups while CG calculations refer to (E-E) beads.

Table S25. Molecular weight of the PIs examined, mean squared end-to-end distance, mean squared radius of gyration and their ratio at 300 K and 1 bar.

| **Polymer** | **Molecular weight (trimer)** (*kDa*) | $\langle \boldsymbol{R^2} \rangle$ (*Å*$^2$) | $\langle \boldsymbol{R_g^2} \rangle$ (*Å*$^2$) | $\frac{\langle \boldsymbol{R^2} \rangle}{\langle \boldsymbol{R_g^2} \rangle}$ |
|---|---|---|---|---|
| **PPD** | 1.55 | 592±5 | 101.5±0.3 | 5.83±0.05 |
| **MPD** | 1.55 | 698±6 | 105.7±0.4 | 6.61±0.06 |
| **25DAT** | 1.60 | 784±6 | 122.9±0.5 | 6.38±0.05 |
| **DPX** | 1.64 | 445±5 | 97.4±0.5 | 4.57±0.06 |
| **DAM** | 1.68 | 613±5 | 100.1±0.3 | 6.12±0.05 |
| **TMPPD** | 1.72 | 531±10 | 98.7±0.5 | 5.38±0.10 |
| **5CMPD** | 1.69 | 452±5 | 95.5±0.4 | 4.73±0.05 |
| **15ND** | 1.66 | 894±6 | 135.8±0.5 | 6.59±0.05 |

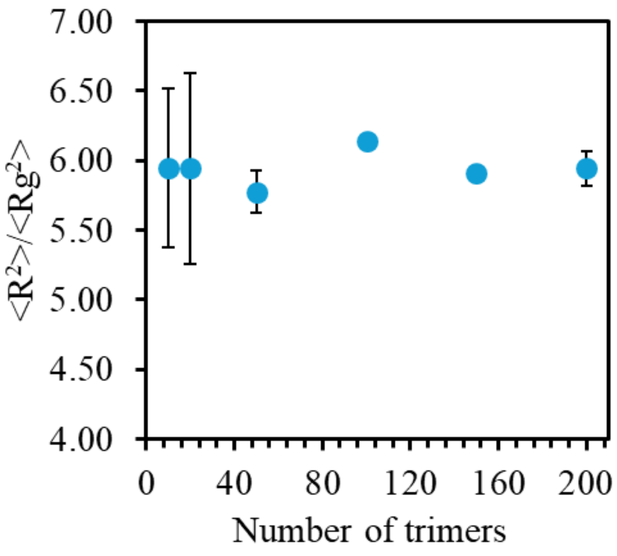


Figure S11. Ratio of the mean squared end-to-end distance to the mean squared radius of gyration for 6FDA-DAM trimer polymer systems as a function of the number of polymer chains. Error bars represent the standard deviation calculated from three independent initial configurations.

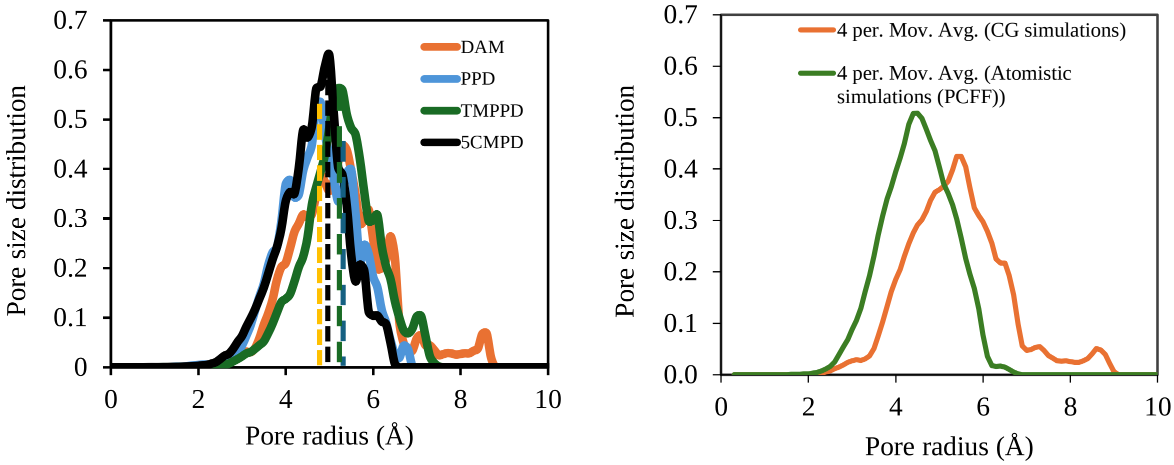


(a) (b)

Figure S12. (a) Pore size distribution comparison between atomistic and CG models for 6FDA-DAM polymer, using a single final structure, (b) pore size distribution of polymeric structure snapshot at 300 K and 1 bar based on the derivative distribution (change of accessible volume to probe size).

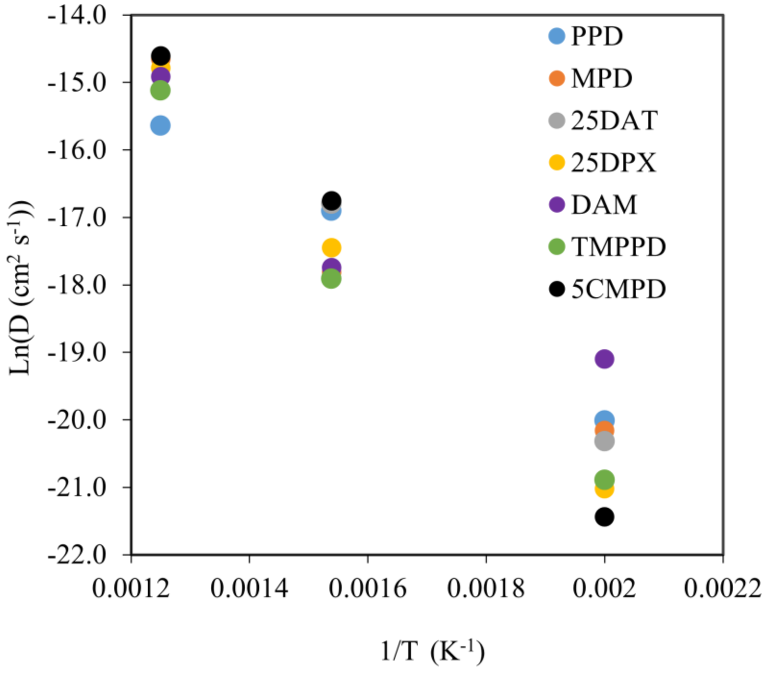


Figure S13. Self-diffusion coefficient of PIs at 500 K, 650 K and 800 K and 1 bar.

## Section 6: Details on GCMC simulations

### 6.1. Detailed elementary move distribution used in CG GCMC simulations

Table S26: Elementary move distribution employed in the GCMC simulations

| Move | $CO_2$, $CH_4$, propane, propylene | | $N_2$, $O_2$ | |
|---|---|---|---|---|
| | polymer | adsorbate | polymer | adsorbate |
| Center of mass translation | 15% | | 20% | |
| Rotation | 15% | | 20% | |
| Regrowth | 20% | 0% | 20% | 0% |
| Insertion | 0% | 25% | 0 | 20% |
| Deletion | 0% | 25% | 0 | 20% |

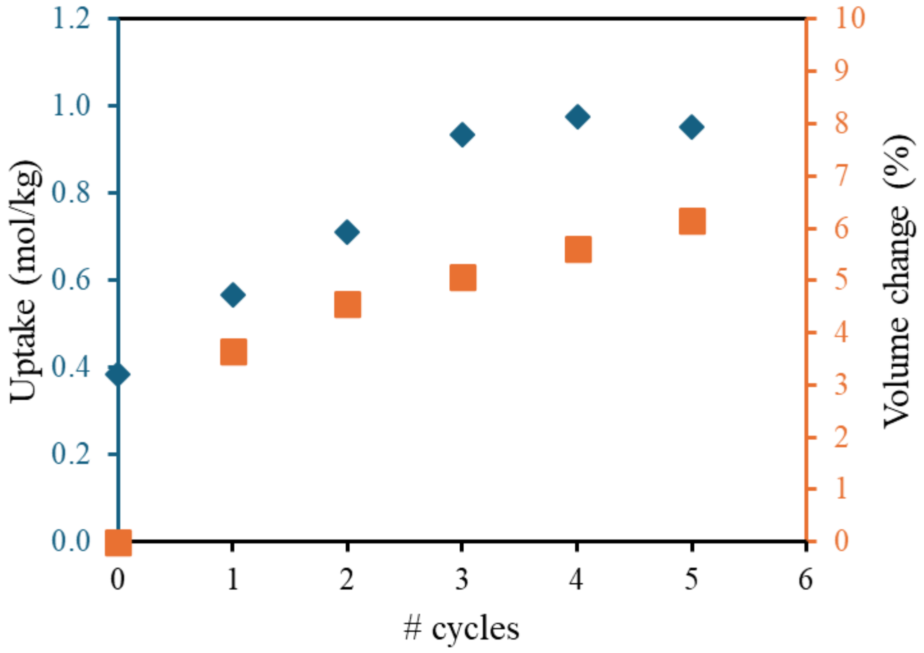


Figure S14. Propylene adsorption at 298 K and 1 bar (left y-axis) and percentage of polymer matrix swelling (right y-axis) as a function of number of cycles.

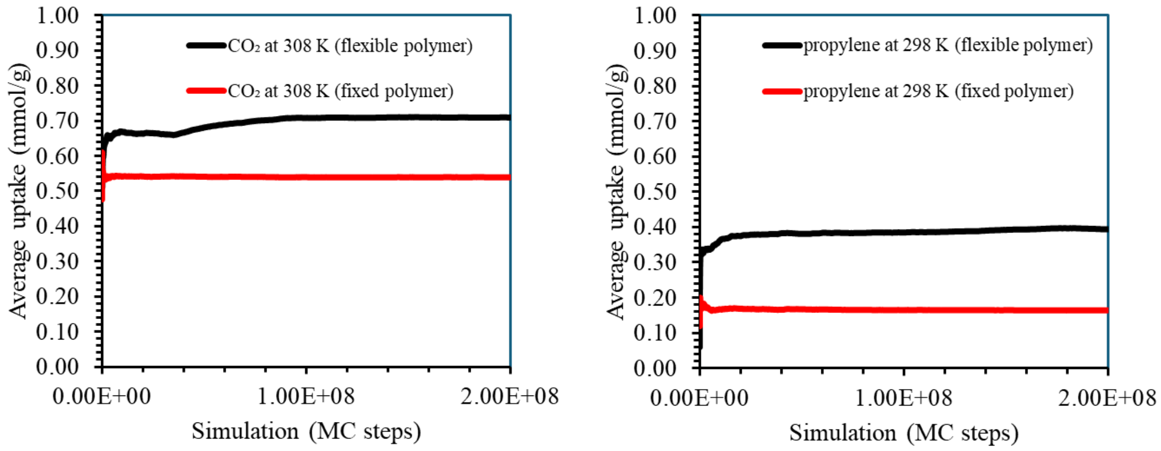


Figure S15. Average uptake of $CO_2$ and propylene as a function of simulation steps in the case of fixed and flexible polymeric chains.

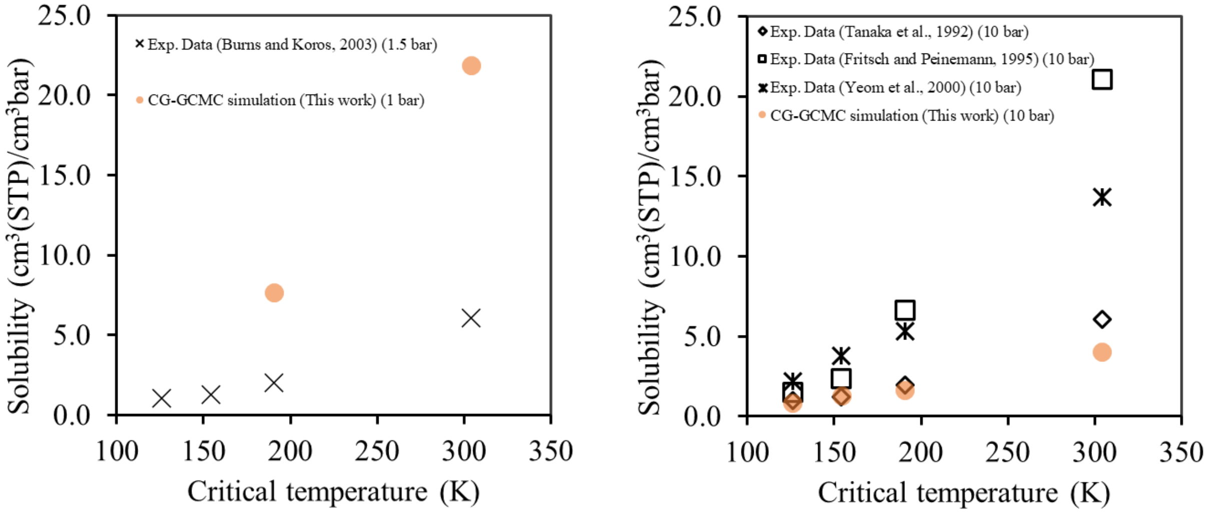


(a) (b)

Figure S16. Experimental data and molecular simulation values for $CO_2$, $CH_4$, $N_2$ and $O_2$ solubility in 6FDA-DAM at 308 K at (a) low pressure, and (b) high pressure as a function of the critical temperature of the gas.

**Section 7: Mechanical property (bulk modulus)**

Table S27: Model parameters for bulk modulus model used in equation 2 in the main text.

| Parameter | value | Standard error |
|---|---|---|
| a | -19.07 | 3.73 |
| b | 33.473 | 7.06 |
| c | 0.0519 | 0.0063 |

## References


(1) Souza, P. C. T.; Alessandri, R.; Barnoud, J.; Thallmair, S.; Faustino, I.; Grünewald, F.; Patmanidis, I.; Abdizadeh, H.; Bruininks, B. M. H.; Wassenaar, T. A.; Kroon, P. C.; Melcr, J.; Nieto, V.; Corradi, V.; Khan, H. M.; Domański, J.; Javanainen, M.; Martinez-Seara, H.; Reuter, N.; Best, R. B.; Vattulainen, I.; Monticelli, L.; Periole, X.; Tieleman, D. P.; de Vries, A. H.; Marrink, S. J. Martini 3: A General Purpose Force Field for Coarse-Grained Molecular Dynamics. *Nat Methods* **2021**, *18* (4), 382–388. https://doi.org/10.1038/s41592-021-01098-3.

(2) Poling, B. E.; Prausnitz, J. M.; O'Connell, J. P. *The Properties of Gases and Liquids*, 5th Edition.; McGraw Hill, 2004.

(3) Shimazu, A.; Miyazaki, T.; Maeda, M.; Ikeda, K. Relationships between the Chemical Structures and the Solubility, Diffusivity, and Permselectivity of Propylene and Propane in 6FDA-Based Polyimides. *J Polym Sci B Polym Phys* **2000**, *38* (19), 2525–2536. https://doi.org/10.1002/1099-0488(20001001)38:19<2525::AID-POLB40>3.0.CO;2-2.
